# Cellulose-Based Ultralong Room-Temperature Phosphorescence Nanomaterials with Tunable Color and High Quantum Yield via Nano-Surface Confining Effect

**DOI:** 10.34133/research.0029

**Published:** 2023-01-30

**Authors:** Xin Zhang, Chunchun Yin, Jingxuan You, Ruiqiao Li, Jinming Zhang, Yaohui Cheng, Yirong Wang, Jun Zhang

**Affiliations:** ^1^ CAS Key Laboratory of Engineering Plastics, CAS Research/Education Center for Excellence in Molecular Sciences, Institute of Chemistry, Chinese Academy of Sciences (CAS), Beijing, 100190, China.; ^2^ University of Chinese Academy of Sciences, Beijing, 100049, China.

## Abstract

How to achieve multicolor organic room-temperature phosphorescence (RTP) is still challenging and striking. Herein, we discovered a new principle to construct eco-friendly color-tunable RTP nanomaterials based on the nano-surface confining effect. Cellulose nanocrystal (CNC) immobilized cellulose derivatives (CX) containing aromatic substituents via hydrogen-bonding interactions, which effectively inhibit the motion of cellulose chains and luminescent groups to suppress the nonradiative transitions. Meanwhile, CNC with a strong hydrogen-bonding network can isolate oxygen. CX with different aromatic substituents regulate the phosphorescent emission. After mixing CNC and CX directly, a series of polychromatic ultralong RTP nanomaterials were obtained. The RTP emission of the resultant CX@CNC can be finely adjusted through the introduction of various CX and the regulation of the CX/CNC ratio. Such a universal, facile, and effective strategy can be used to fabricate various colorful RTP materials with wide color gamut. Because of the complete biodegradability of cellulose, the multicolor phosphorescent CX@CNC nanomaterials can be used as eco-friendly security inks to fabricate disposable anticounterfeiting labels and information-storage patterns via conventional printing and writing processes.

## Introduction

Pure organic room-temperature phosphorescence (RTP) materials have attracted tremendous attention recently because of their huge potential in information storage and encryption [1–3], biological imaging [4–6], optical display [7–9], etc. Two basic principles of designing RTP materials must be obeyed, including the effective intersystem crossing (ISC) and the suppression of nonradiative transitions [10–12]. Until today, most of the reported organic RTP materials are crystalline organic small molecules, exhibiting the limited processability and poor formability [13]. In contrast, polymer-based RTP materials have excellent processability and formability. But there are few intrinsic RTP polymers at present [14–18]. Polymers are generally used as the matrix to confine the movement of phosphors, such as polyvinyl alcohol [19], polymethyl methacrylate [20], polyacrylamide [21], etc. It is appealing and valuable in practice to discover new construction principles, develop more facile preparation methods, and enrich the polymer-based RTP materials.

Compared with the single-color phosphorescence, multicolor phosphorescence can hide or load more abundant information. If the time-dependent emission and dual luminescence (fluorescence and phosphorescence) of RTP materials are considered, massive information can be stored in a colorful phosphorescence pattern. However, the preparation of colorful RTP materials is still challenging. Colorful phosphorescent materials are divided into 2 categories according to the luminescence principle at present. The first category is the RTP materials that have different luminescence centers with different excitation wavelengths. Such colorful RTP materials with multiple luminescence centers generally have excitation-dependent phosphorescence emission or time-dependent phosphorescence lifetime. However, the phosphorescence emission with strong dependence on the excitation wavelength or time is not easy to be precisely adjusted, and it is difficult to cover the whole visible-light spectrum [22–26]. Recently, Wang et al. [23] synthesized the triazine derivatives, whose phosphorescence emission could be adjusted from green to purple dynamically by changing the excitation wavelength. In addition, they also introduced different phosphorescent centers on the polymer chain to prepare RTP materials with tunable phosphorescence emission from blue to yellow [24]. The second category is the RTP materials with different chemical structures or compositions. Such colorful RTP materials exhibit stable phosphorescence emission and cover a wider range of phosphorescence emission that can be precisely adjusted [1,27–30]. Under the same excitation wavelength, colorful phosphorescent display can be realized. Lei et al. [1] prepared color-tunable RTP materials with phosphorescence emission from cyan to orange red by changing the guest molecules, based on the host-guest strategy. Dou et al. [31] introduced different phosphors onto the sodium alginate chains to prepare RTP materials with tunable phosphorescence emission from blue to orange red.

Cellulose is the most abundant biopolymer with extensive sources in nature [32–35]. Because there are plenty of hydroxyl groups along the polymer chain, cellulose has strong hydrogen-bonding networks, just like the polyvinyl alcohol and polyacrylamide, which are the most efficient matrix of the organic RTP materials [15,36]. Therefore, cellulose can be considered as an excellent natural RTP matrix material [37,38]. Gong et al. [39] first discovered that natural polysaccharides such as cellulose and starch had weak RTP performance. The clusters that were formed by cellulose chains through hydrogen-bonding interactions promoted ISC; meanwhile, the movement of cellulose chains was confined by the strong hydrogen-bonding interactions. The phosphorescence of pure cellulose is obviously weakened after treatment with NaOH [40]. Zeng et al. [41] constructed a series of photoenhanced RTP materials with excellent luminescent and mechanical properties based on the intermolecular hydrogen-bonding interactions of cellulose. This is because the natural cellulose has the most perfect hydrogen-bonding network, and the hydrogen-bonding interactions in cellulose can be destroyed once cellulose is treated with NaOH. These phenomena further prove that the strong hydrogen-bonding network is extremely important in the preparation of cellulose-based RTP materials. Recently, we found that cellulose-based ultralong RTP materials could be prepared via introducing phosphors into cellulose chains meanwhile enhancing interchain interactions [42,43]. Cellulose nanocrystal (CNC) is a natural cellulose I crystal, which possesses the strongest hydrogen-bonding interactions and the perfect hydrogen-bonding network. Furthermore, CNC exhibits a series of outstanding physical and chemical properties, such as nanoscale size, high specific surface area, low density, high mechanical strength, biocompatibility, and biodegradability [44–46]. Thus, CNC is an ideal matrix to prepare phosphorescent materials. However, cellulose has weak phosphorescence intensity and single phosphorescent color. The introduction of phosphors will destroy the perfect hydrogen-bonding network, resulting in the decline of phosphorescence performance. Therefore, how to utilize the natural nanomaterial CNC to construct RTP materials is extremely striking.

**Fig. 1.**
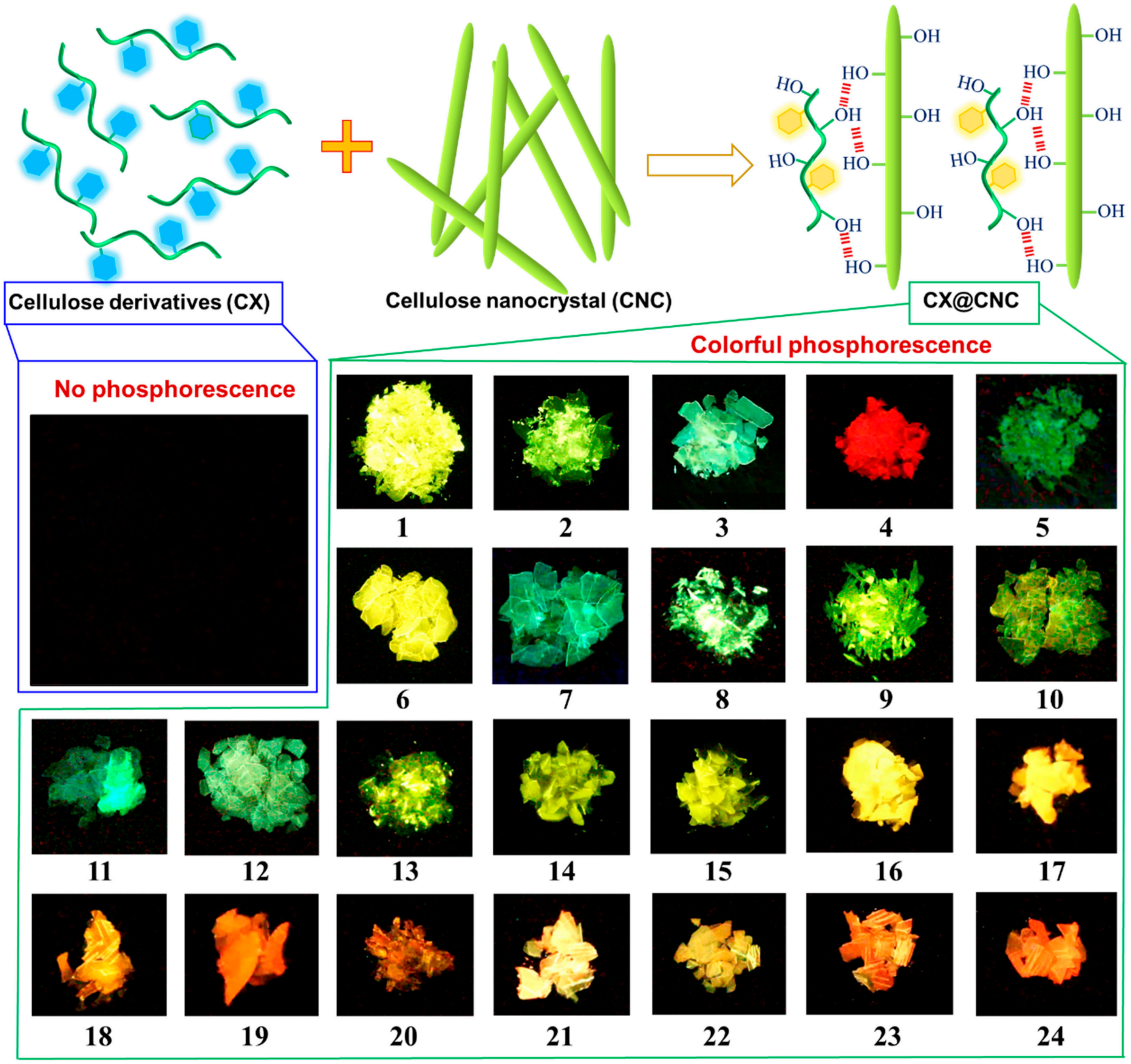
Schematic illustration and phosphorescent photographs of cellulose-based colorful RTP materials CX@CNC. (The detail information of CX@CNC system is shown in Table S1.)

In this work, we proposed and demonstrated a new principle to construct a series of cellulose-based colorful RTP nanomaterials via immobilizing cellulose-based phosphors on the surface of CNC based on the hydrogen-bonding interactions (Fig. 1). The resultant RTP nanomaterials have tunable phosphorescent emission ranging from cyan (499 nm) to red (626 nm). They can be used as printable and writable security inks for advanced anticounterfeiting, information handling, organic electronics, and so on.

## Results

### Preparation and properties of cellulose-based colorful RTP materials

**Fig. 2.**
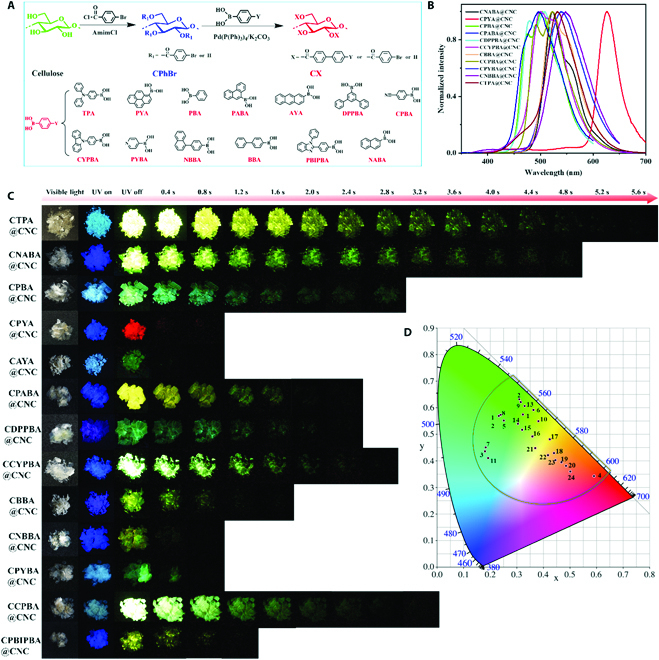
Preparation and properties of cellulose-based colorful RTP materials CX@CNC. (A) Synthesis route of CX. (B) Normalized phosphorescence spectra of CX@CNC. (C) Photographs of CX@CNC taken under the UV lamp and with the lamp off. (D) Commission Internationale d'Eclairage coordinate diagram of CX@CNC. (The UV lamp for CTPA@CNC, CPYA@CNC, CCYPBA@CNC, and CPBIPBA@CNC is 365 nm, and the UV lamp for the rest of samples is 313 nm.) (The detail information of CX@CNC system for Fig. 2C and D is shown in Table S1.)

CNC is the needle or rod-shaped nanofiber with a diameter of 3 to 10 nm and a length of 50 to 500 nm. CNC has numerous hydroxyl groups on the surface. When CNC is mixed with cellulose derivatives (CX) containing aromatic groups, the hydroxyl groups on the surface of CNC can form hydrogen-bonding interactions with the hydroxyl groups of CX. The CX is immobilized on the surface of CNC, inhibiting its movement to realize RTP (Fig. 1). The CX acts as the phosphorescent luminophore. Via adjusting the chemical structure of the substituent on CX, colorful RTP materials can be regulated (Fig. 2). This method is effective, feasible, and universal to construct RTP materials via confining cellulose-based phosphorescent luminophores on CNC surface.

The CX was prepared by a 2-step homogeneous derivatization reaction. Firstly, cellulose 4-bromobenzoate (CPhBr) was synthesized by an esterification of cellulose in an ionic liquid 1-allyl-3-methylimidazolium chloride (AmimCl). Subsequently, the CPhBr reacted with phenylboronic acid derivatives to obtain CX containing large conjugated groups by Suzuki coupling reaction (Fig. 2A). In proton nuclear magnetic resonance (^1^H-NMR) spectrum of the intermediate CPhBr (Fig. S1A), the peak at 7.2 to 8.0 ppm is assigned to the protons on the benzene ring, and the peak at 2.8 to 5.5 ppm is the protons of cellulose backbone. In the Fourier transform infrared (FTIR) spectrum (Fig. S1B), the new peak at 1716 cm^−1^ is the carbonyl stretching vibration peak. According to the ^1^H-NMR spectrum, the degree of substitution (DS) of 4-bromobenzoate in CPhBr can be calculated to be 1.42. Further, after a Suzuki coupling reaction of CPhBr, CX that contained different aromatic substituents and DS_X_ values was prepared (Figs. S2 to S15) [47]. For example, in the ^1^H-NMR spectrum of cellulose 4-triphenylamine benzoate (CTPA), the peak intensity of triphenylamine (TPA) at 6.7 to 7.2 ppm increases gradually with the increase of the DS of TPA (Fig. S2A). According to the ^1^H-NMR spectrum, the DS of TPA can be calculated (Table S2). In the FTIR spectrum of CTPA, the characteristic peaks of the benzene ring at 1489, 827, 754, and 696 cm^−1^ gradually enhance as the DS of TPA increases (Fig. S2B). X-ray photoelectron spectroscopy (XPS) curves indicate that CPhBr is composed of C, O, and Br elements, while CTPA is composed of C, O, Br, and N elements (Fig. S3). These results confirm that the cellulose ester CTPA containing a large conjugated 4-triphenylaminophenyl group was successfully prepared.

Subsequently, the CX/*N*,*N*′-dimethylformamide (DMF) solution (0.1 mol/l) and the CNC/DMF solution (1.64 wt%) were mixed, and then DMF was removed by heating to obtain CX@CNC RTP nanomaterials. The obtained CX@CNC exhibits excellent RTP properties. For instance, CTPA@CNC emits sky-blue fluorescence when it is irradiated with a 365-nm ultraviolet (UV) lamp and emits yellow phosphorescence after the UV lamp is turned off. The phosphorescence lasts for more than 5 s at room temperature (Fig. 2B to D). By changing the chemical structure of the conjugated substituent on CX, CX@CNC exhibits a different phosphorescence emission and lifetime. The phosphorescence emission of CX@CNC can be tuned from 499 nm of CPYBA@CNC to 626 nm of CPYA@CNC, realizing the adjustment of phosphorescent colors from cyan to red (Fig. 2B to D). Moreover, after mixing different kinds of CX@CNC, we can further precisely adjust the phosphorescent colors, which cover the green and red intervals in the Commission Internationale d'Eclairage diagram (Fig. 2B to D and Table S1).

### Mechanism of cellulose-based colorful RTP materials

**Fig. 3.**
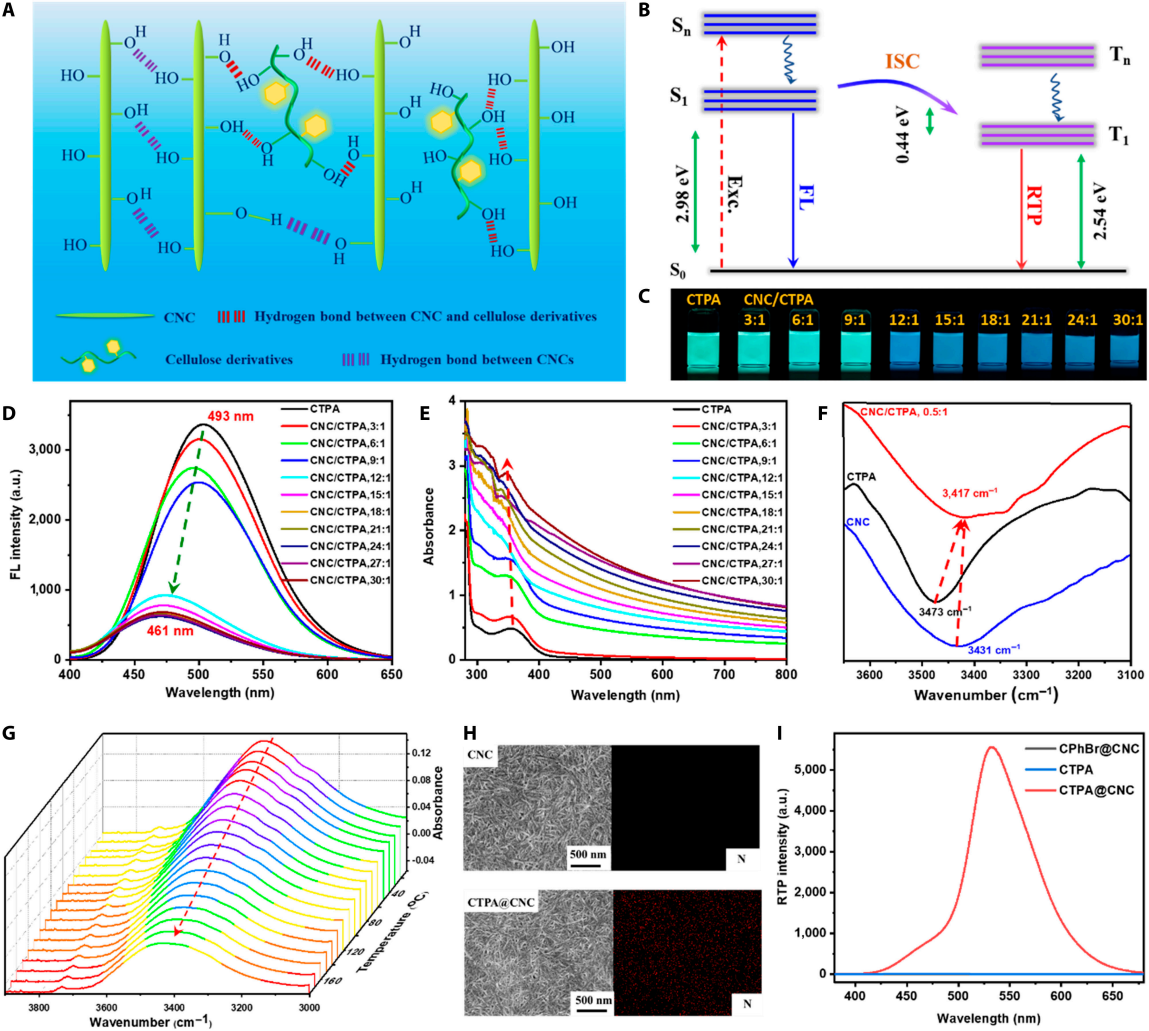
Mechanism of cellulose-based colorful RTP materials. (A) Schematic diagram of the phosphorescence mechanism of CX@CNC. (B) Jablonski diagram energy level diagram of CX@CNC, and the energy gap belongs to CTPA@CNC. (C) Photographs of CNC/CTPA/DMF solution with different mass ratios of CNC/CTPA taken under 365-nm UV light. (D) Fluorescence (FL) spectra of CNC/CTPA/DMF solution with different mass ratios of CNC/CTPA (Ex = 365 nm). a.u., arbitrary units. (E) Absorption spectra of CNC/CTPA/DMF solution with different mass ratios of CNC/CTPA. (F) FTIR spectra of CTPA, CNC, and CTPA@CNC (CNC:CTPA = 0.5:1). (G) Variable temperature FTIR spectra of CTPA@CNC (CNC:CTPA = 5:1). (H) Scanning electron microscopy and energy-dispersive spectroscopy images of CNC and CTPA@CNC (CNC:CTPA = 5:1). (I) RTP spectra of CTPA, CTPA@CNC (CNC:CTPA = 5:1; DS_TPA_ = 0.12), and CPhBr@CNC (CNC:CPhBr = 5:1) (Ex = 370 nm).

The hydrogen-bonding interactions between the hydroxyl groups on the surface of CNC and the hydroxyl groups of CX effectively restrict the movement of CX chain and aromatic substituent on CX to suppress the nonradiative transitions (Fig. 3A). The CNC with the strong hydrogen-bonding network can isolate oxygen [48–51]. The 4-bromobenzoate substituent and large conjugated substituent on the CX promote ISC (Fig. 3B). As a result, CX@CNC exhibits phosphorescent emission at room temperature. Taking CTPA@CNC for example, the RTP mechanism was demonstrated. When the CTPA concentration was fixed, the fluorescence spectra of the CNC/CTPA/DMF solutions obviously changed as the increase of CNC concentration. When the CNC/CTPA ratio was more than 12:1 (w/w), the fluorescence color changed from cyan (493 nm) to blue (461 nm), and the fluorescence intensity decreased substantially (Fig. 3C and D). Moreover, UV absorption intensity of the CNC/CTPA/DMF dispersion gradually increased with the increase of CNC content (Fig. 3E). The changes of fluorescence color, fluorescence intensity, and UV absorption intensity of CNC/CTPA/DMF indicate that there is an intermolecular interaction between CTPA and CNC. Then, we replaced CNC/DMF dispersion with cellulose diacetate (CDA)/DMF solution and found that as the CDA/CTPA ratio increased, the fluorescence emission wavelength, fluorescence emission intensity, and UV absorption of the corresponding CDA/CTPA/DMF solutions had a negligible change (Fig. S16). Compared with CNC, CDA with a high DS of acetate substituent contains few hydroxyl groups, thus the hydrogen-bonding interactions between CDA and CTPA are weak. The above results further confirm that there is a strong interaction between CTPA and CNC. Compared with the hydroxyl stretching vibration peaks of CNC (3431 cm^−1^) and CTPA (3473 cm^−1^), the O-H stretching vibration of CNC/CTPA (0.5:1) gives a remarkable shift to 3417 cm^−1^ (Fig. 3F). Moreover, when the temperature increases from 30 to 200 °C, the hydroxyl stretching vibration peak of CNC/CTPA (5:1, w/w) shifts from 3423 to 3435 cm^−1^, and its intensity also changes, indicating that CNC and CTPA form hydrogen-bonding interactions (Fig. 3G). Scanning electron microscopy and energy-dispersive spectroscopy images show that CTPA is adsorbed on the surface of CNC (Fig. 3H). The CTPA has no impact on the crystal structure of CNC due to the surface adsorption (Fig. S17). Thus, via the hydrogen-bonding interactions, CTPA is adsorbed on the surface of CNC, which effectively inhibits the movement of CTPA.

The CX/DMF solutions have bright phosphorescence at 77 K. The emission color is controlled by the substituent group (X). For example, the phosphorescence color of CTPA is green, the phosphorescence color of CPYA is red, and the phosphorescence color of CCYPBA is cyan at 77 K (Fig. S18). The CNC powder exhibits only green phosphorescence with very weak luminescence intensity at room temperature (Fig. S19). The CX powder has no phosphorescence at room temperature (Fig. 3I). Once the CX is composited with CNC, the resultant CX@CNC can exhibit a strong RTP performance (Figs. 2B to D and 3I). If CPhBr is composited with CNC, the obtained CPhBr@CNC has a negligible RTP (Fig. 3I). Thus, CX containing 4-bromobenzoate substituent and a large conjugated substituent can be considered as the phosphorescent luminophores, and the change of the X group can control the phosphorescence color.

In summary, cellulose-based phosphor CX was immobilized on the surface of CNC through hydrogen-bonding interactions, which suppressed the nonradiative transitions of the triplet excited state. Via controlling the conjugated substituent, a series of cellulose-based color-tunable organic RTP materials were successfully prepared.

### Performance regulation of cellulose-based colorful RTP materials

**Fig. 4.**
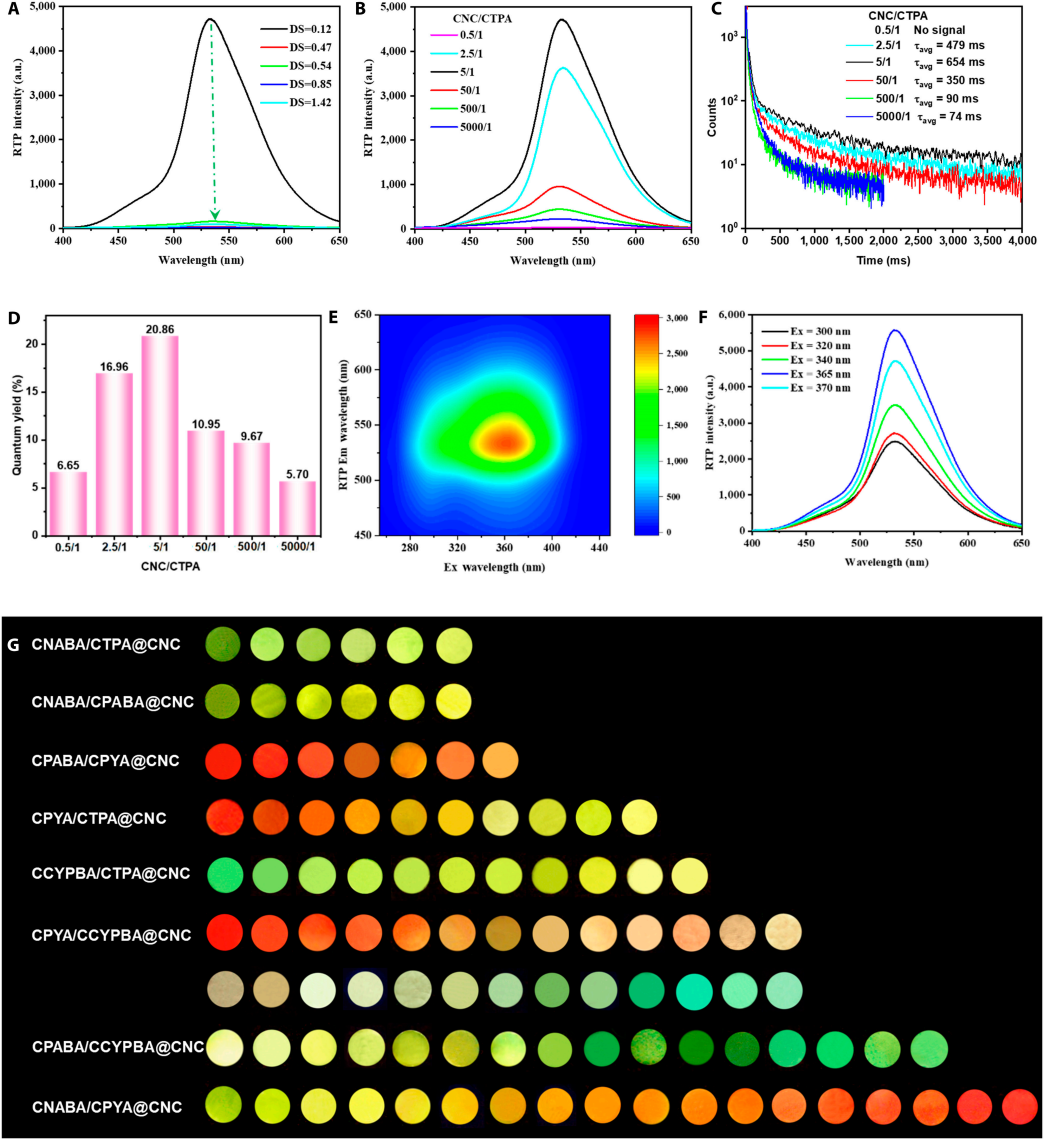
RTP performance of CTPA@CNC. (A) RTP spectra of CTPA@CNC corresponding to CTPA with different DS (CNC/CTPA = 5:1 (w/w); Ex = 370 nm). (B) RTP spectra of CTPA@CNC corresponding to CNC/CTPA with different mass ratios (DS_TPA_ = 0.12). (C) RTP lifetime spectra of CTPA@CNC corresponding to CNC/CTPA with different mass ratios (DS_TPA_ = 0.12). (D) Photoluminescence quantum yield of CTPA@CNC corresponding to CNC/CTPA with different mass ratios (DS_TPA_ = 0.12; Ex = 370 nm). (E) Excitation-phosphorescence emission mapping of CTPA@CNC (DS_TPA_ = 0.12; CNC/CTPA = 5:1). (F) RTP spectra of CTPA@CNC (DS_TPA_ = 0.12; CNC/CTPA = 5:1) under different excitation wavelengths. (G) Phosphorescence colors of CX@CNC containing 2 kinds of cellulose derivatives. (The molar ratio of CPABA/CPYA, CNABA/CTPA and CNABA/CPABA are from 10/1 to 1/1, while the others are from 10/1 to 1/10.)

The RTP properties of the resultant CX@CNC RTP materials can be altered by tuning the chemical structure of CX and the CNC/CX ratio. Taking CTPA for example, we fixed the mass ratio of CNC/CTPA at 5:1 and changed the DS_TPA_. It is found that, when the DS_TPA_ is 0.12, the CTPA@CNC exhibits the highest phosphorescence intensity, which is much higher than those of other samples (Fig. 4A and Fig. S20). This is because the formation of hydrogen-bonding interactions between CTPA and CNC is hindered by the bulky 4-triphenylamine benzoate group as the DS_TPA_ increases. In addition, the fluorescence intensity of CTPA@CNC gradually decreases with the increase of the DS_TPA_, indicating that the aggregation-induced quenching behavior of CTPA@CNC is another reason for the decline of their RTP performance (Fig. S20). Subsequently, we increased the mass ratio of CNC/CTPA to enhance the nanosurface confining effect and found that when the mass ratio of CNC/CTPA reached to 500:1, the CTPA@CNC with the CTPA of DS = 0.85 exhibited the best phosphorescence performance (Fig. S21). For the CTPA with a DS of 0.47, the CTPA@CNC has the optimal phosphorescence performance when the mass ratio of CNC/CTPA is 50:1 (Fig. S22). For the CTPA with a DS of 0.54, the CTPA@CNC has the optimal phosphorescence performance when the mass ratio of CNC/CTPA is 500:1 (Fig. S22). However, as the increase of the mass ratio of CNC/CTPA, the phosphorescence intensity of CTPA@CNC decreases significantly. Comprehensively considering the DS_TPA_ and the CNC/CTPA ratio, when the DS_TPA_ is 0.12 and the CNC/CTPA mass ratio is 5:1, CTPA@CNC has the best phosphorescence performance with an average phosphorescence lifetime of 654 ms and a photoluminescence quantum yield as high as 20.86% (Fig. 4B to D and Tables S3 and S4). For different substituent groups (X), different CNC/CX ratios (12:1 to 1,200:1) are required to achieve the optimal RTP properties (Figs. S23 and S34 and Tables S1 to S4). For example, when the mass ratio of CNC/CX is 50:1, CPIPBA@CNC and CCYPBA@CNC have the best phosphorescence performance, and their photoluminescence quantum yields are as high as 24.61% and 34.00%, respectively.

It should be noticed that most of the obtained CX@CNC exhibit a large difference between fluorescence and phosphorescence emission, which are beneficial to avoid the background interference and fluorescence self-quenching. For instance, the difference between the fluorescence emission wavelength and phosphorescence emission wavelength of CTPA@CNC and CNABA@CNC materials is about 70 and 130 nm, respectively (Figs. S20 and S23).

The phosphorescence emission wavelength of CX@CNC, such as CTPA@CNC, does not change significantly with the change of excitation wavelength, indicating that there is no excitation-dependent phosphorescence emission in CX@CNC (Fig. 4E and F). Therefore, the phosphorescence emission of CX@CNC relies on the exciton of the conjugated substituent (X group), rather than the cluster emission. The phosphorescence color can be controlled by changing the structure of X group. In addition, we can mix 2 kinds of CX to further regulate the phosphorescence color. Via adjusting the chemical structure of CX and the CNC/CX ratio, a series of RTP materials with different phosphorescence emission can be obtained (Fig. 4G).

### Applications of cellulose-based colorful RTP materials

**Fig. 5.**
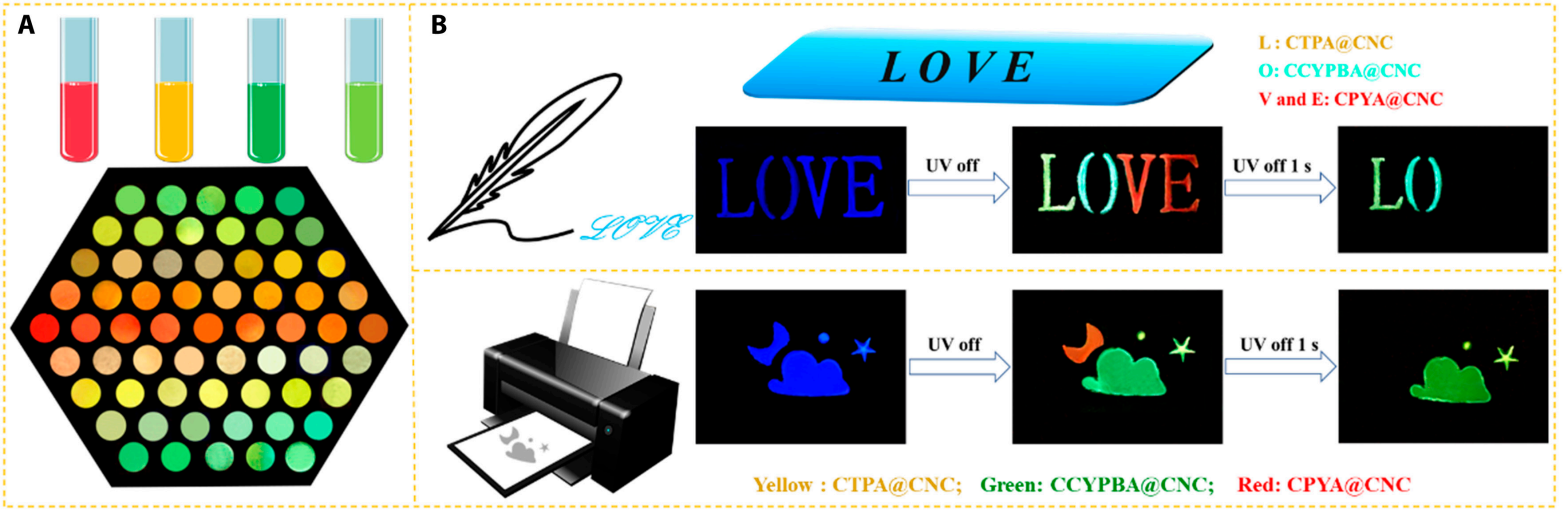
Phosphorescent inks of CX@CNC and their applications. (A) Phosphorescent inks of CX@CNC with different colors. (B) Colorful phosphorescent patterns (Ex = 365 nm).

The obtained CX@CNC maintains the needle-shaped nanostructure (Fig. 3H and S35), which is the same as the shape and size of CNC. Thus, the CX@CNC RTP nanomaterials can be used as the nano-ink or nano-filler for anticounterfeiting patterns and information storage by simple processing methods, such as inkjet printing, screen printing, and template technology. We mixed different CX@CNC to obtain a series of phosphorescent inks with different colors (Fig. 5). Three different colors of phosphorescent inks, including green (CCYPBA@CNC), yellow (CTPA@CNC), and red (CPYA@CNC), were selected for writing and printing (Fig. 5B). Under the 365-nm UV lamp, “LOVE” shows blue fluorescence. When the UV lamp is turned off, the colorful “LOVE” word appears. After the UV lamp is turned off for 1 s, the red “VE” disappears, and only “LO” is remained. Additionally, these 3 phosphorescent inks were used to print the colorful phosphorescent patterns. The pattern shows a dynamic change as the observation time is prolonged (Fig. 5B). The colorful, dynamic, and multiemission patterns can store massive information and exhibit advanced anticounterfeiting effect.

## Conclusion

We discovered and demonstrated a new method to construct RTP materials based on the nanosurface confining effect on CNC. A series of cellulose-based eco-friendly color-tunable organic RTP nanomaterials were obtained. CNC confined the movement of cellulose derivative chains and phosphors through hydrogen-bonding interactions, meanwhile isolating oxygen. Cellulose derivative CX worked as the phosphorescent luminophores. Such a new method is simple, effective, and universal. The obtained CX@CNC exhibited long RTP lifetime and high quantum yield, which is up to 34.00%. Via manipulating the chemical structures of CX and the CX/CNC ratios, the precise and fine regulation of phosphorescence emission from cyan to red was achieved. The CX@CNC could be fully biodegraded because the main component is cellulose. Such novel phosphorescent nanomaterials were used as eco-friendly colorful security inks, indicating a huge potential in disposable advanced anticounterfeiting labels and information-storage patterns.

## Supplementary Materials

Supplementary 1 Experimental section Figs. S1 to S35 Tables S1 to S4
