# Supplementary material for: Cellulose-Based Ultralong Room-Temperature Phosphorescence Nanomaterials with Tunable Color and High Quantum Yield via Nano-Surface Confining Effect
Source: Research (Wash D C). 2023 Jan 30;6:0029. doi: 10.34133/research.0029 (PMC10076006; doi:10.34133/research.0029)
Supplement: Supplementary 1 — Experimental section Figs. S1 to S35 Tables S1 to S4 [file research.0029.f1.docx]

**Supporting Information**

**Cellulose-based Ultralong Room-Temperature Phosphorescence Nanomaterials with Tunable Color and High Quantum Yield via Nano-Surface Confining Effect**

Xin Zhang^1,2^, Chunchun Yin^1,2^, Jingxuan You^1,2^, Ruiqiao Li^1^, Jinming Zhang^1,^*, Yaohui Cheng^1,2^, Yirong Wang^1,2^, Jun Zhang^1,2^

^1^ CAS Key Laboratory of Engineering Plastics, CAS Research/Education Center for Excellence in Molecular Sciences, Institute of Chemistry, Chinese Academy of Sciences (CAS), Beijing, 100190, China.

^2^ University of Chinese Academy of Sciences, Beijing, 100049, China.

**Supporting information content**

Number of pages: 33

Number of figures: 35

Number of tables: 4

**Experimental section**

**Materials**

Cellulose (microcrystalline cellulose, PH-101) with an average degree of polymerization (DP) of 220 was purchased from Beijing Fengli Jingqiu Commerce and Trade Company (China). It was dried in vacuum at 80 °C for 24 h. Cellulose diacetate (CDA) with a substitution degree of 2.45 was purchased from Sichuan Push Acetati Co., Ltd. (China), and was dried in vacuum at 80 °C for 24 h. Cellulose nanocrystal (CNC) with a diameter of 3-10 nm and a length of 50-500 nm was purchased from ScienceK Co., Ltd. (China). 4-Bromobenzoyl chloride, potassium carbonate (K_2_CO_3_), tetrakis(triphenylphosphine)palladium (Pd(P(Ph)_3_)_4_), 4-cyanophenylboronic acid (CPBA), phenylboronic acid (PBA), 4-biphenylboronic acid (BBA), 4-(naphthalen-1-yl)phenylboronic acid (NBBA), (3,5-diphenylphenyl)boronic acid (DPPBA), (4-(diphenylamino)phenylboronic acid (TPA), (4-(9H-carbazol-9-yl)phenyl)boronic acid (CYPBA), pyridin-4-ylboronic acid (PYBA), anthracen-2-ylboronic acid (AYA), 1-pyreneboronic acid (PYA), 2-naphthaleneboronic acid (NABA), 9-phenanthreneboronic acid (PABA) and (4-(1-phenyl-1H-benzo[d]imidazol-2-yl)phenyl)boronic acid (PBIPBA) were purchased from Innochem and J&K Scientific. Ultra-dry tetrahydrofuran (THF) with a moisture content below 30 ppm was obtained from Innochem. N,N'-dimethylformamide (DMF) and ethanol were received from Tianjin Concord Technology Co., Ltd. Concentrated hydrochloric acid was purchased from Sinopharm Chemical Reagent Co., Ltd. (China). The dialysis bag with a molecular weight cut-off of 3500 was purchased from Beijing Ruida Henghui Technology Development Co., Ltd. (China). 1-Allyl-3-methylimidazolium chloride (AmimCl) was synthesized in the laboratory [1]. The water content in AmimCl determined by Karl Fischer method was less than 0.3 wt%. The double-distilled water (>18.2 MΩ cm^-1^) from the Millipore Milli-Q system was used in all experiments.

**Measurements**

^1^H-NMR spectra were acquired on a Bruker AV-400 NMR spectrometer with 16 scans at room temperature in DMSO-d_6_. A 20-μL aliquot of CF_3_COOH-d_1_ was added to shift the signals of the free hydrogens to the downfield. FTIR spectra was recorded with a Nicolet 6700 FT-IR spectrometer (Thermo Fisher, USA) from 650 to 4000 cm^-1^ with 32 scans. X-ray photoelectron spectroscopy (XPS) was performed on a Thermo Scientific ESCALab 250Xi spectrometer (USA) using 200 W monochromatic Al Kα radiation. Fluorescence spectra and phosphorescence spectra were recorded with a Hitachi F-7000 fluorescence spectrophotometer (Japan). The photoluminescence quantum yield and phosphorescence lifetime were measured on an Edinburgh FLS980 steady-state transient fluorescence spectrometer (UK) with an integrating sphere and a microsecond flash lamp. The phosphorescence lifetime was measured by multi-channel single photon technology. The UV-vis absorption spectra was recorded with a Shimadzu UV-2600 spectrometer (Japan). Fluorescence and phosphorescence images were captured with a digital camera (SONY α7, Japan). The SEM and EDS images were recorded with a Hitachi SU8020 field emission scanning electron microscope (Japan) with an accessory of energy dispersive spectrometer (EDS). The TEM image was recorded with a JEM-1011 transmission electron microscope (Japan).

**Synthesis of cellulose derivatives**

Via a homogeneous synthesis process, the following cellulose derivatives were synthesized: cellulose para-bromobenzoate (CPhBr), cellulose 4-triphenylamine benzoate (CTPA), cellulose 4-(1-pyrene)benzoate (CPYA), cellulose 4-phenylbenzoate (CPBA), cellulose 4-(9-phenanthrene)benzoate (CPABA), cellulose 4-(anthracen-2-yl)benzoate (CAYA), cellulose 4-(3,5-diphenylphenyl)benzoate (CDPPBA), cellulose 4-(4-cyanophenyl)benzoate (CCPBA), cellulose 4-(4-(9H-carbazol-9-yl)phenyl))benzoate (CCYPBA), cellulose 4-(pyridin-4-yl)benzoate (CPYBA), cellulose 4-(4-biphenyl)benzoate (CBBA), cellulose 4-(4-(1-phenyl-1H-benzo[d]imidazol-2-yl)phenyl)benzoate (CPBIPBA), cellulose 4-(4-(naphthalen-1-yl)phenyl)benzoate (CNBBA) and cellulose 4-(2-naphthalene)benzoate (CNABA).

**Synthesis of CPhBr.** Cellulose (6 g, 37.04 mmol) was completely dissolved in 114 g of the ionic liquid AmimCl at 80 °C. After cellulose was completely dissolved, 4-bromobenzoyl chloride (24.88 g, 111.10 mmol) and K_2_CO_3_ (7.68 g, 55.56 mmol) were added to the cellulose/AmimCl solution at 70 °C for 3 h. Ethanol was added to the reaction system to remove unreacted 4-bromobenzoyl chloride. Then the reaction solution was precipitated in a mixed solvent of ethanol/water (v/v = 1/1). The crude product was collected by filtration. The product was washed three times with ethanol and two times with water. Finally, the product was filtered and dried under vacuum at 60 °C for 24 h before characterization.

**Synthesis of CTPA.** The intermediate CPhBr (DS_Br_ = 1.42, 844 mg, 2.00 mmol) was dissolved in 15 mL of ultra-dry THF. Then, Pd(P(Ph)_3_)_4_ (16 mg-195 mg, 0.013 mmol-0.168 mmol), K_2_CO_3_ (111 mg-1382 mg, 0.80 mmol-10.00 mmol) and TPA (118 mg-1475 mg, 0.40 mmol-5.0 mmol) were added to the CPhBr/THF solution at 60 °C for 24 h under N_2_. The product was precipitated in ethanol/water (v/v = 1/1), and collected by filtration. The product was washed three times with ethanol and two times with water. Finally, the product was filtered and dried under vacuum at 60 °C for 24 h before characterization.

**Synthesis of CPYA.** The intermediate CPhBr (DS_Br_ = 1.42, 844 mg, 2.00 mmol) was dissolved in 15 mL of ultra-dry THF. Then, Pd(P(Ph)_3_)_4_ (16 mg, 0.013 mmol), K_2_CO_3_ (111 mg, 0.80 mmol) and PYA (104 mg, 0.40 mmo) were added to the CPhBr/THF solution at 60 °C for 12 h under N_2_. The product was precipitated in ethanol/water (v/v = 1/1), and collected by filtration. The product was washed three times with ethanol and two times with water. Finally, the product was filtered and dried under vacuum at 60 °C for 24 h before characterization.

**Synthesis of CPBA.** The intermediate CPhBr (DS_Br_ = 1.42, 844 mg, 2.00 mmol) was dissolved in 15 mL of ultra-dry THF. Then, Pd(P(Ph)_3_)_4_ (16 mg, 0.013 mmol), K_2_CO_3_ (111 mg, 0.80 mmol) and PBA (50 mg, 0.40 mmol) were added to the CPhBr/THF solution at 60 °C for 12 h under N_2_. The product was precipitated in ethanol/water (v/v = 1/1), and collected by filtration. The product was washed three times with ethanol and two times with water. Finally, the product was filtered and dried under vacuum at 60 °C for 24 h before characterization.

**Synthesis of CPABA.** The intermediate CPhBr (DS_Br_ = 1.42, 844 mg, 2.00 mmol) was dissolved in 15 mL of ultra-dry THF. Then, Pd(P(Ph)_3_)_4_ (16 mg, 0.013 mmol), K_2_CO_3_ (111 mg, 0.80 mmol) and PABA (91 mg, 0.40 mmol) were added to the CPhBr/THF solution at 60 °C for 12 h under N_2_. The product was precipitated in ethanol/water (v/v = 1/1), and collected by filtration. The product was washed three times with ethanol and two times with water. Finally, the product was filtered and dried under vacuum at 60 °C for 24 h before characterization.

**Synthesis of CAYA.** The intermediate CPhBr (DS_Br_ = 1.42, 844 mg, 2.00 mmol) was dissolved in 15 mL of ultra-dry THF. Then, Pd(P(Ph)_3_)_4_ (16 mg, 0.013 mmol), K_2_CO_3_ (111 mg, 0.80 mmol) and AYA (91 mg, 0.40 mmol) were added to the CPhBr/THF solution at 60 °C for 12 h under N_2_. The product was precipitated in ethanol/water (v/v = 1/1), and collected by filtration. The product was washed three times with ethanol and two times with water. Finally, the product was filtered and dried under vacuum at 60 °C for 24 h before characterization.

**Synthesis of CDPPBA.** The intermediate CPhBr (DS_Br_ = 1.42, 844 mg, 2.00 mmol) was dissolved in 15 mL of ultra-dry THF. Then, Pd(P(Ph)_3_)_4_ (16 mg, 0.013 mmol), K_2_CO_3_ (111 mg, 0.80 mmol) and DPPBA (98 mg, 0.40 mmol) were added to the CPhBr/THF solution at 60 °C for 12 h under N_2_. The product was precipitated in ethanol/water (v/v = 1/1), and collected by filtration. The product was washed three times with ethanol and two times with water. Finally, the product was filtered and dried under vacuum at 60 °C for 24 h before characterization.

**Synthesis of CCPBA.** The intermediate CPhBr (DS_Br_ = 1.42, 844 mg, 2.00 mmol) was dissolved in 15 mL of ultra-dry THF. Then, Pd(P(Ph)_3_)_4_ (16 mg, 0.013 mmol), K_2_CO_3_ (111 mg, 0.80 mmol) and CPBA (60 mg, 0.40 mmol) were added to the CPhBr/THF solution at 60 °C for 12 h under N_2_. The product was precipitated in ethanol/water (v/v = 1/1), and collected by filtration. The product was washed three times with ethanol and two times with water. Finally, the product was filtered and dried under vacuum at 60 °C for 24 h before characterization.

**Synthesis of CCYPBA.** The intermediate CPhBr (DS_Br_ = 1.42, 844 mg, 2.00 mmol) was dissolved in 15 mL of ultra-dry THF. Then, Pd(P(Ph)_3_)_4_ (16 mg, 0.013 mmol), K_2_CO_3_ (111 mg, 0.80 mmol) and CYPBA (118 mg, 0.40 mmol) were added to the CPhBr/THF solution at 60 °C for 12 h under N_2_. The product was precipitated in ethanol/water (v/v = 1/1), and collected by filtration. The product was washed three times with ethanol and two times with water. Finally, the product was filtered and dried under vacuum at 60 °C for 24 h before characterization.

**Synthesis of CPYBA.** The intermediate CPhBr (DS_Br_ = 1.42, 844 mg, 2.00 mmol) was dissolved in 15 mL of ultra-dry THF. Then, Pd(P(Ph)_3_)_4_ (16 mg, 0.013 mmol), K_2_CO_3_ (111 mg, 0.80 mmol) and PYBA (44 mg, 0.40 mmol) were added to the CPhBr/THF solution at 60 °C for 12 h under N_2_. The product was precipitated in ethanol/water (v/v = 1/1), and collected by filtration. The product was washed three times with ethanol and two times with water. Finally, the product was filtered and dried under vacuum at 60 °C for 24 h before characterization.

**Synthesis of CBBA.** The intermediate CPhBr (DS_Br_ = 1.42, 844 mg, 2.00 mmol) was dissolved in 15 mL of ultra-dry THF. Then, Pd(P(Ph)_3_)_4_ (16 mg, 0.013 mmol), K_2_CO_3_ (111 mg, 0.80 mmol) and BBA (81 mg, 0.40 mmol) were added to the CPhBr/THF solution at 60 °C for 12 h under N_2_. The product was precipitated in ethanol/water (v/v = 1/1), and collected by filtration. The product was washed three times with ethanol and two times with water. Finally, the product was filtered and dried under vacuum at 60 °C for 24 h before characterization.

**Synthesis of CPBIPBA.** The intermediate CPhBr (DS_Br_ = 1.42, 844 mg, 2.00 mmol) was dissolved in 15 mL of ultra-dry THF. Then, Pd(P(Ph)_3_)_4_ (16 mg, 0.013 mmol), K_2_CO_3_ (111 mg, 0.80 mmol) and PBIPBA (128 mg, 0.40 mmol) were added to the CPhBr/THF solution at 60 °C for 12 h under N_2_. The product was precipitated in ethanol/water (v/v = 1/1), and collected by filtration. The product was washed three times with ethanol and two times with water. Finally, the product was filtered and dried under vacuum at 60 °C for 24 h before characterization.

**Synthesis of CNBBA.** The intermediate CPhBr (DS_Br_ = 1.42, 844 mg, 2.00 mmol) was dissolved in 15 mL of ultra-dry THF. Then, Pd(P(Ph)_3_)_4_ (16 mg, 0.013 mmol), K_2_CO_3_ (111 mg, 0.80 mmol) and NBBA (101 mg, 0.40 mmol) were added to the CPhBr/THF solution at 60 °C for 12 h under N_2_. The product was precipitated in ethanol/water (v/v = 1/1), and collected by filtration. The product was washed three times with ethanol and two times with water. Finally, the product was filtered and dried under vacuum at 60 °C for 24 h before characterization.

**Synthesis of CNABA.** The intermediate CPhBr (DS_Br_ = 1.42, 844 mg, 2.00 mmol) was dissolved in 15 mL of ultra-dry THF. Then, Pd(P(Ph)_3_)_4_ (16 mg, 0.013 mmol), K_2_CO_3_ (111 mg, 0.80 mmol) and NABA (70 mg, 0.40 mmol) were added to the CPhBr/THF solution at 60 °C for 12 h under N_2_. The product was precipitated in ethanol/water (v/v = 1/1), and collected by filtration. The product was washed three times with ethanol and two times with water. Finally, the product was filtered and dried under vacuum at 60 °C for 24 h before characterization.

**Preparation of CNC/DMF dispersion.** CNC (8 g) was added to 800 mL of ultrapure water. The mixture was stirred magnetically for 40 min at room temperature, and then was treated by ultrasonic cell disintegrator (Scientz-IID) for about 40 min. Subsequently, 80 mL of concentrated hydrochloric acid was added to the CNC/water dispersion. After mechanical stirring for 60 min, the dispersion was transferred to a dialysis bag, and was dialyzed twice with ethanol and twice with DMF. The CNC/DMF solution in the dialysis bag was transferred into a beaker, and was treated by ultrasonic cell disintegrator for about 40 min. The CNC/DMF dispersion was centrifuged at 3000 rpm for 10 min to remove undispersed CNC. The mass fraction of CNC in the CNC/DMF dispersion was determined by the differential weight method.

**Preparation of CX@CNC materials.** Single-component or multi-component CX/DMF solution (0.1 mol/L) and CNC/DMF were compounded with different mass ratios. Multi-component CX/DMF solution contained two kinds of cellulose derivatives with different molar ratios. The molar ratio of CPABA/CPYA, CNABA/CTPA and CNABA/CPABA are from 10/1 to 1/1, while the others are from 10/1 to 1/10 in Fig. 4g. The CX/CNC/DMF mixture was treated by ultrasonic for more than 40 min to obtain the CX/CNC/DMF dispersion. Via a doctor blade coating method, a phosphorescent film was obtained. Via screen printing, inkjet printing, and mask casting methods, phosphorescent patterns were formed.


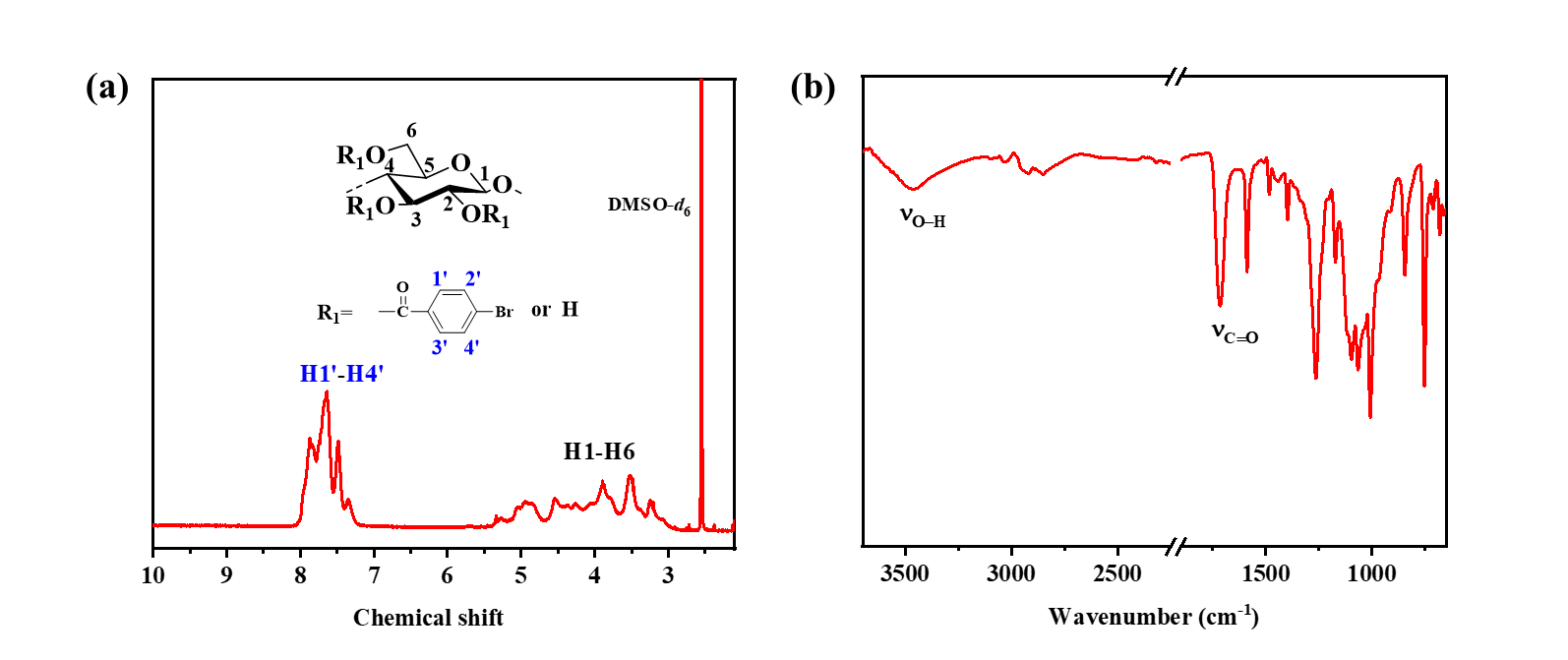


**Figure S1.** (a) ^1^H-NMR spectrum and (b) FTIR spectrum of CPhBr.


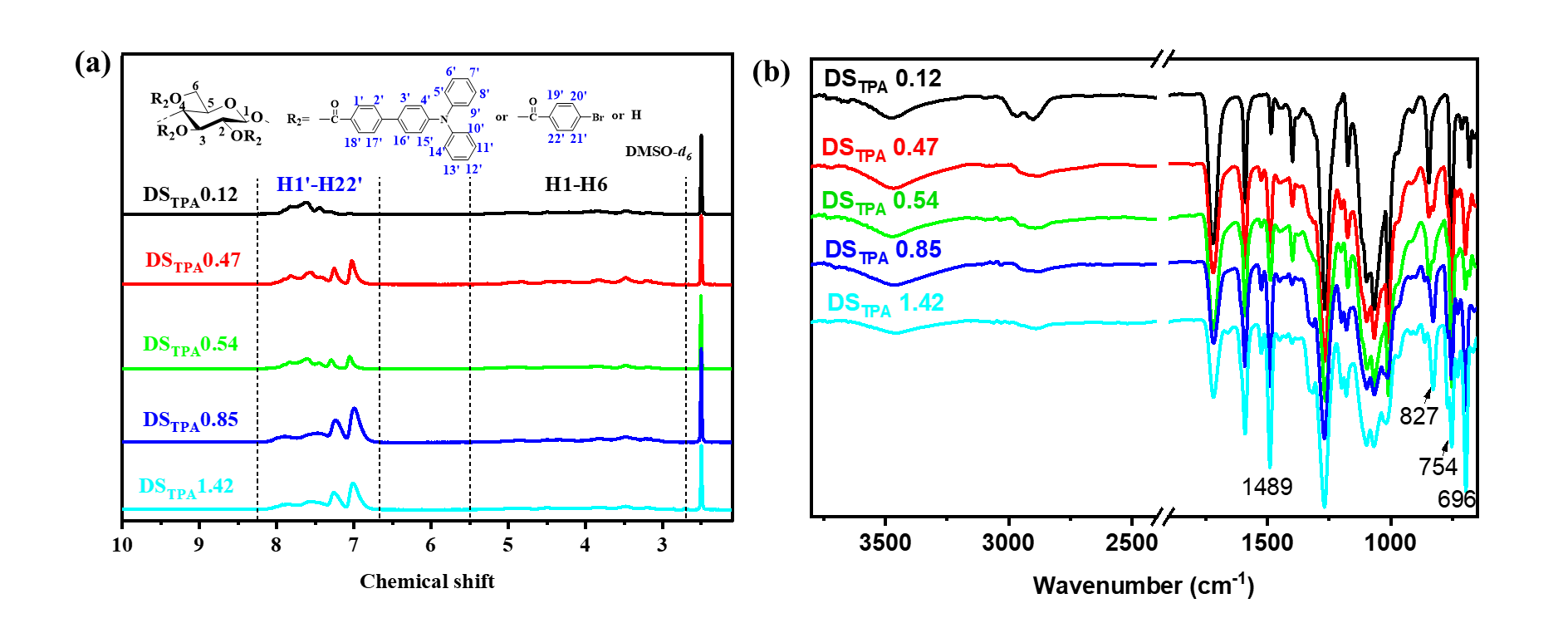


**Figure S2.** (a) ^1^H-NMR spectrum and (b) FTIR spectrum of CTPA.

**Figure S3.** XPS curves of CPhBr and CTPA.


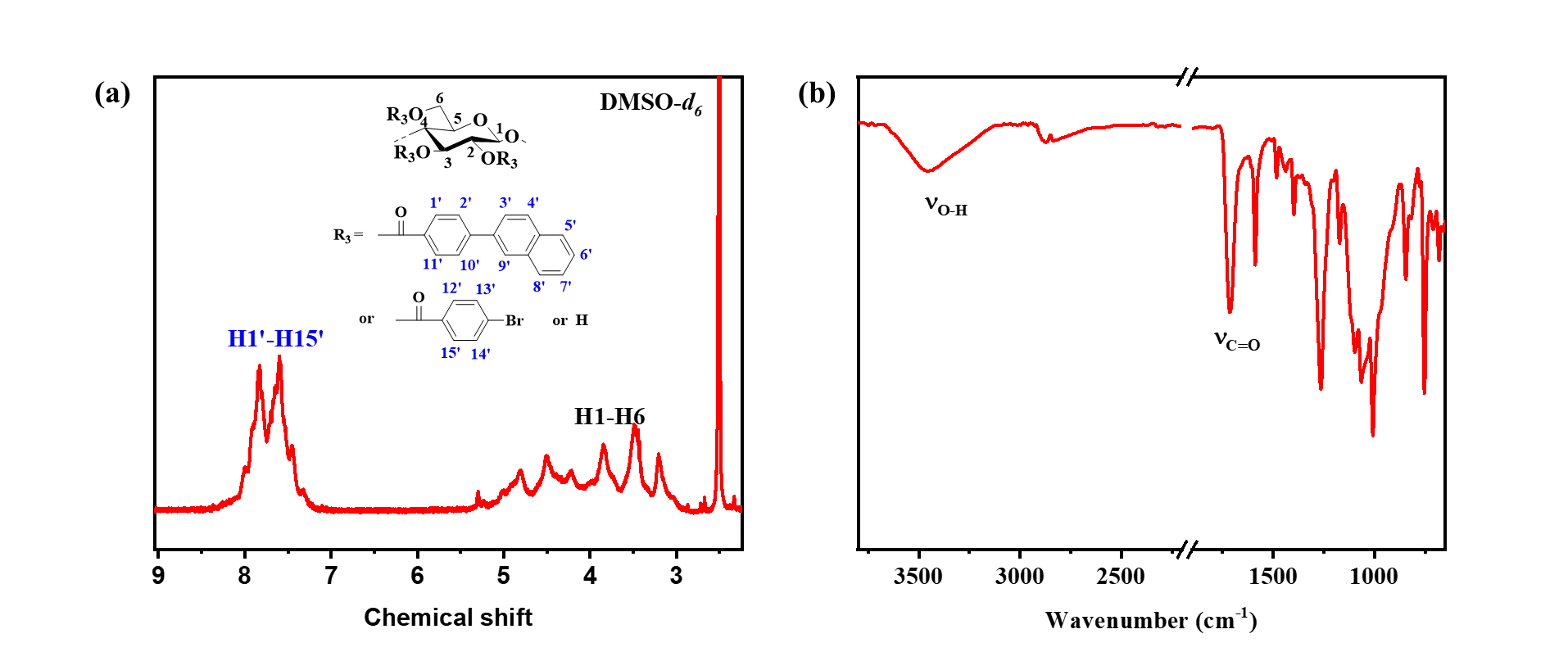


**Figure S4.** (a) ^1^H-NMR spectrum and (b) FTIR spectrum of CNABA.


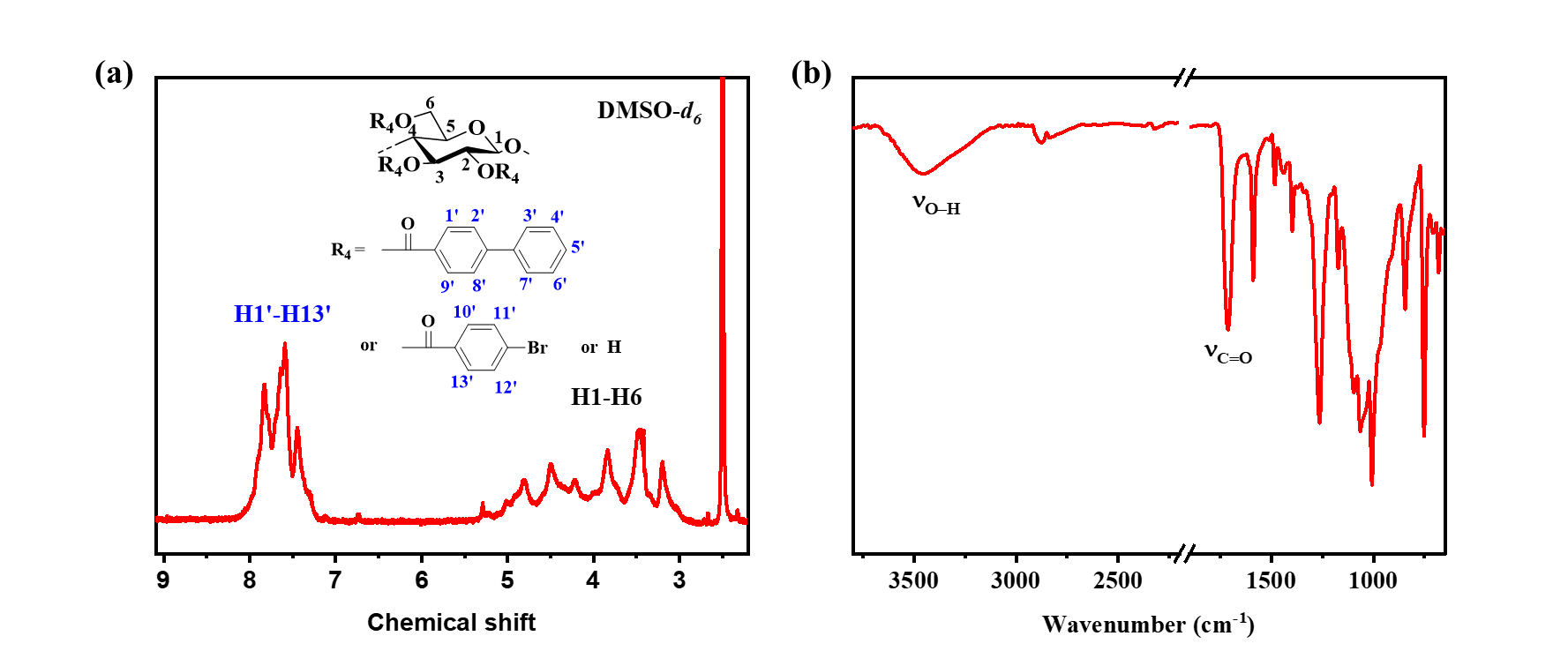


**Figure S5.** (a) ^1^H-NMR spectrum and (b) FTIR spectrum of CPBA.


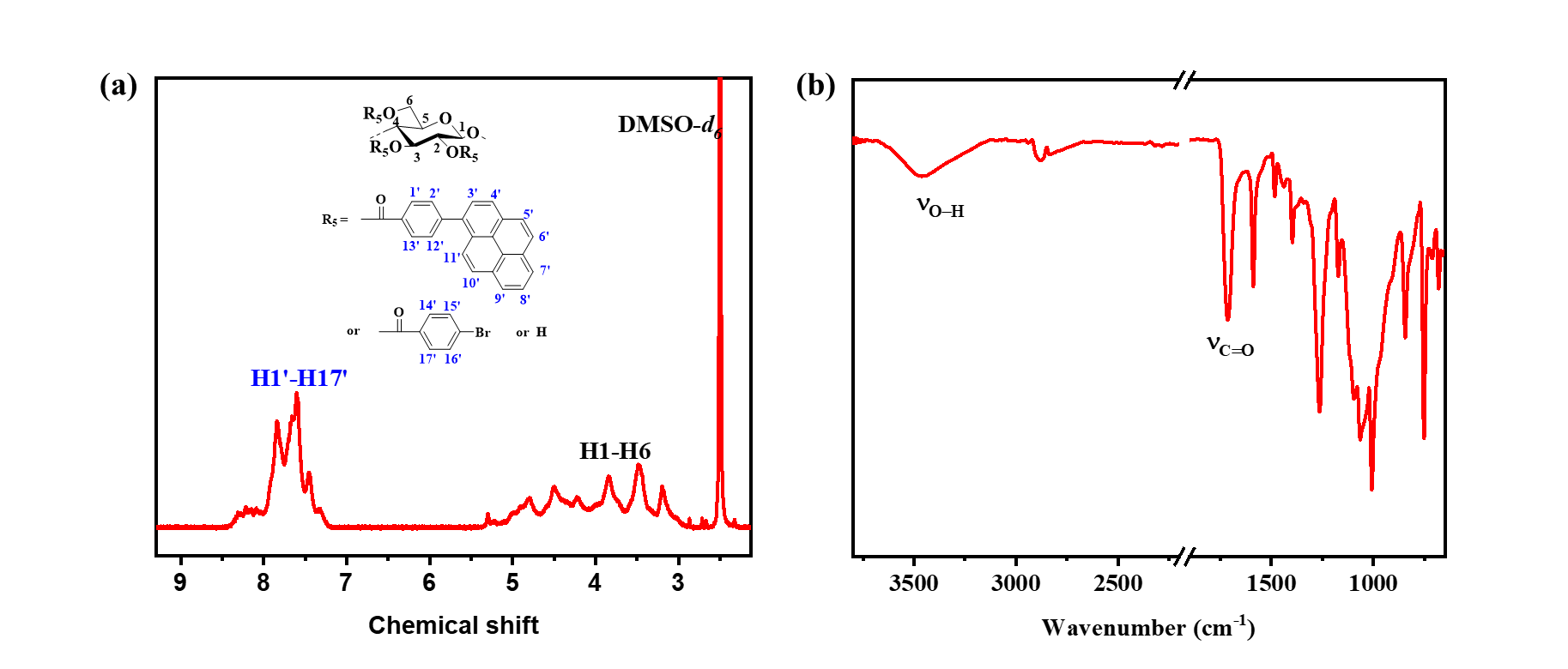


**Figure S6.** (a) ^1^H-NMR spectrum and (b) FTIR spectrum of CPYA.


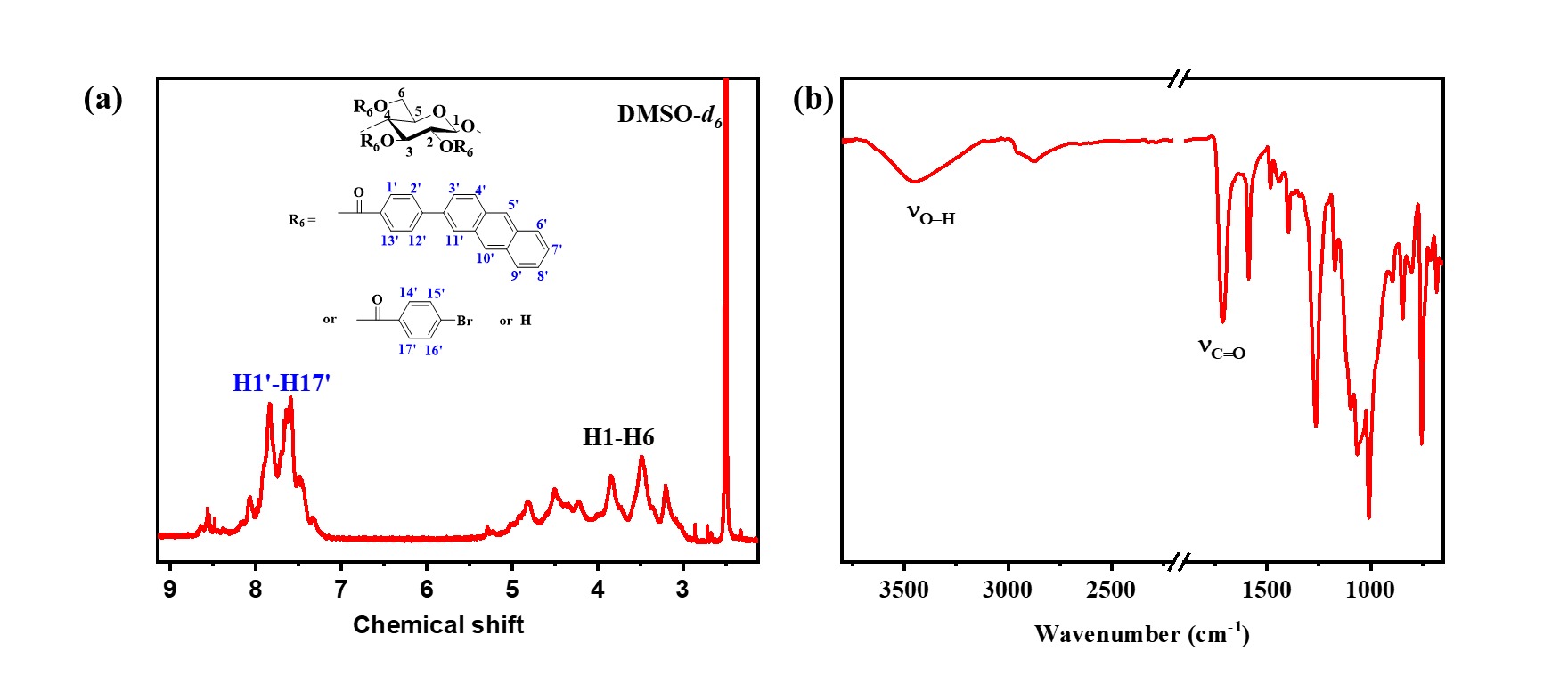


**Figure S7.** (a) ^1^H-NMR spectrum and (b) FTIR spectrum of CAYA.


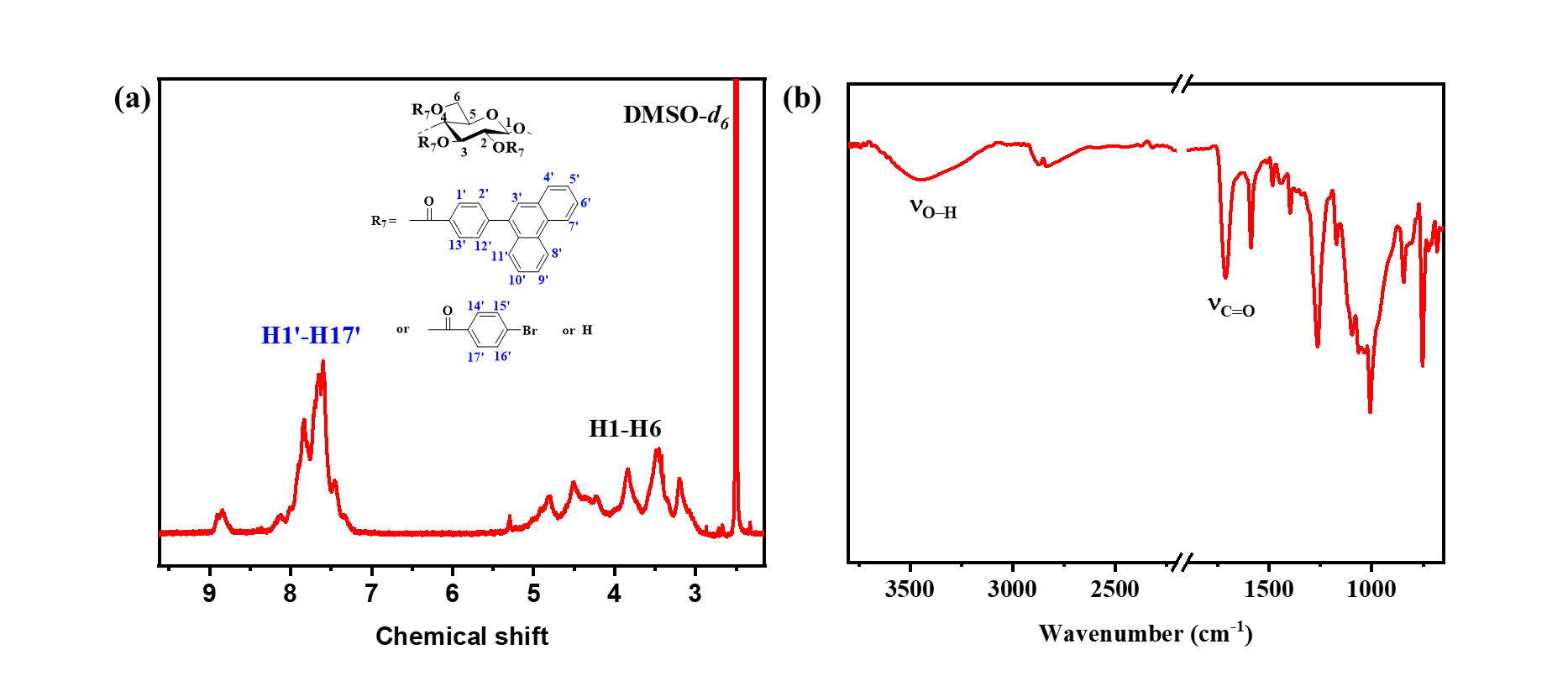


**Figure S8.** (a) ^1^H-NMR spectrum and (b) FTIR spectrum of CPABA.


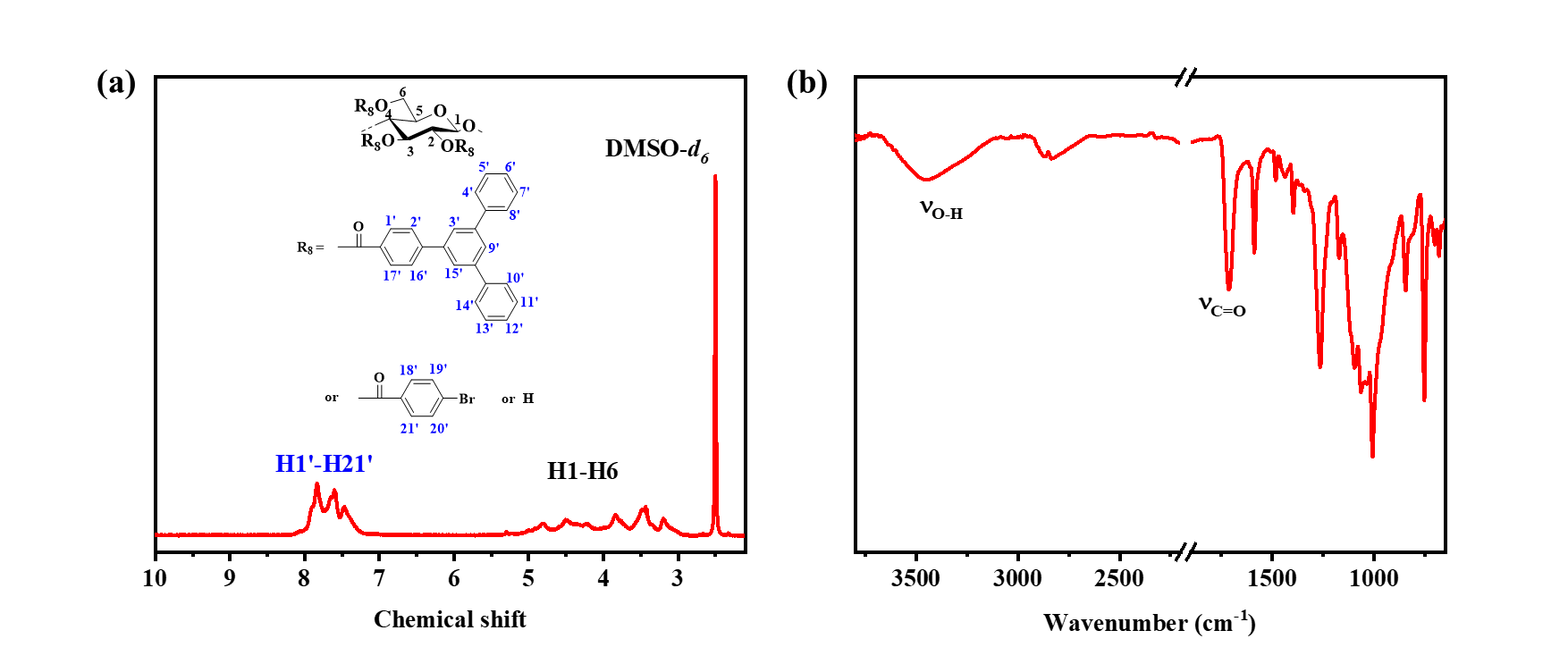


**Figure S9.** (a) ^1^H-NMR spectrum and (b) FTIR spectrum of CDPPBA.


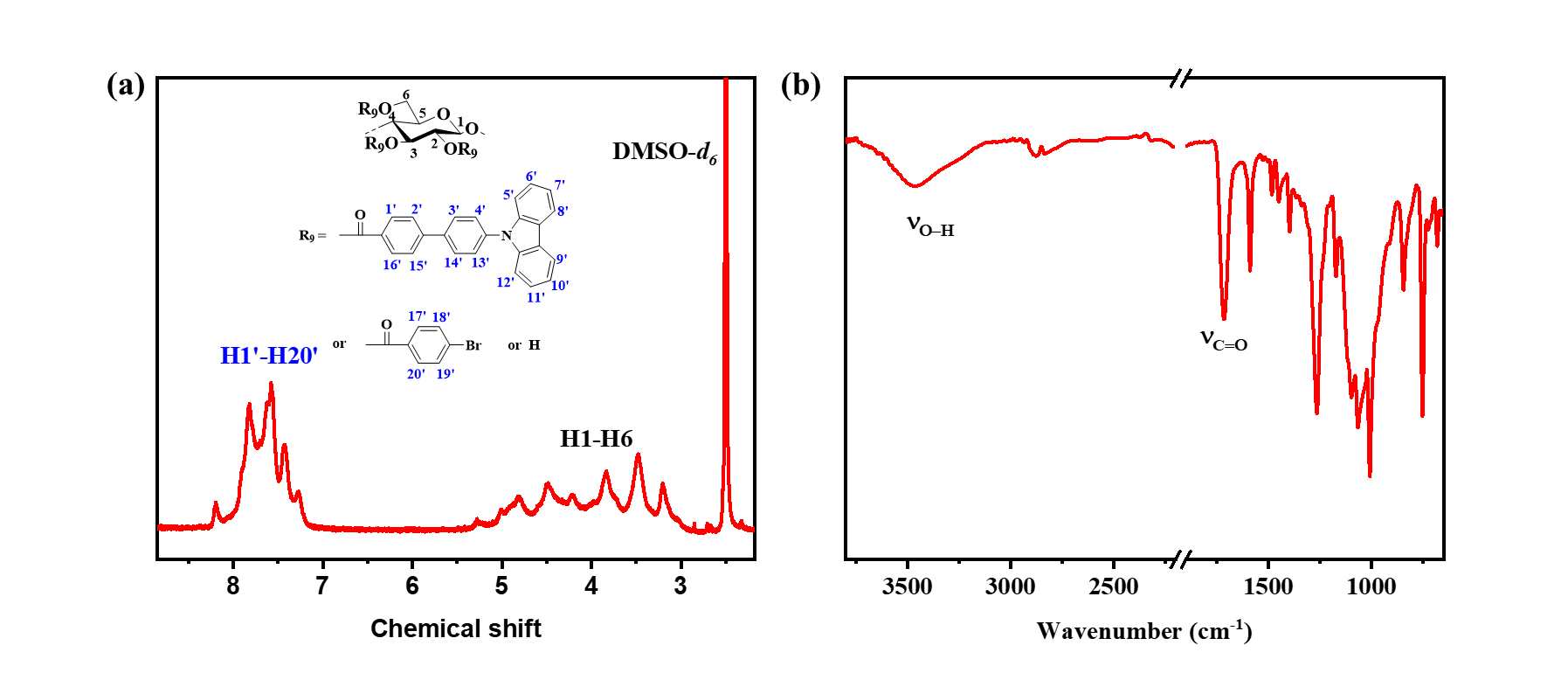


**Figure S10.** (a) ^1^H-NMR spectrum and (b) FTIR spectrum of CCYPBA.


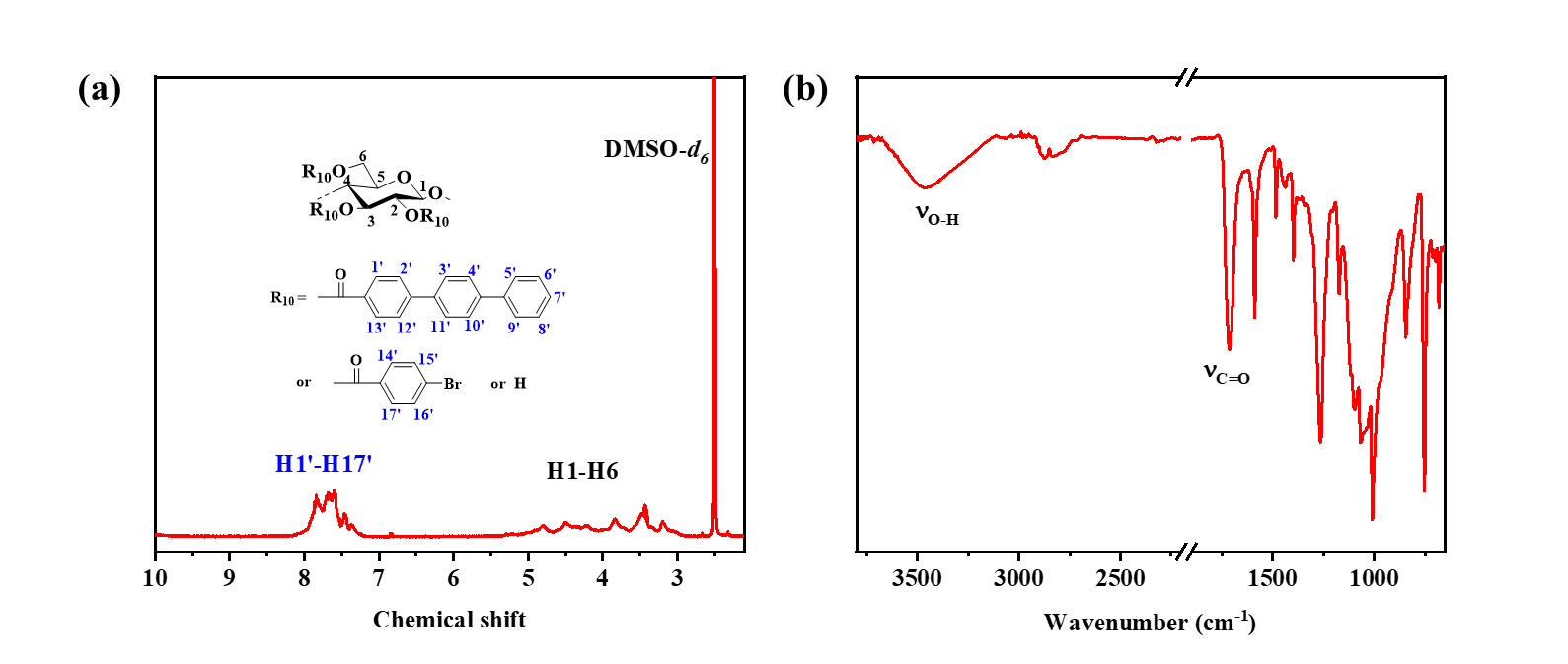


**Figure S11.** (a) ^1^H-NMR spectrum and (b) FTIR spectrum of CBBA.


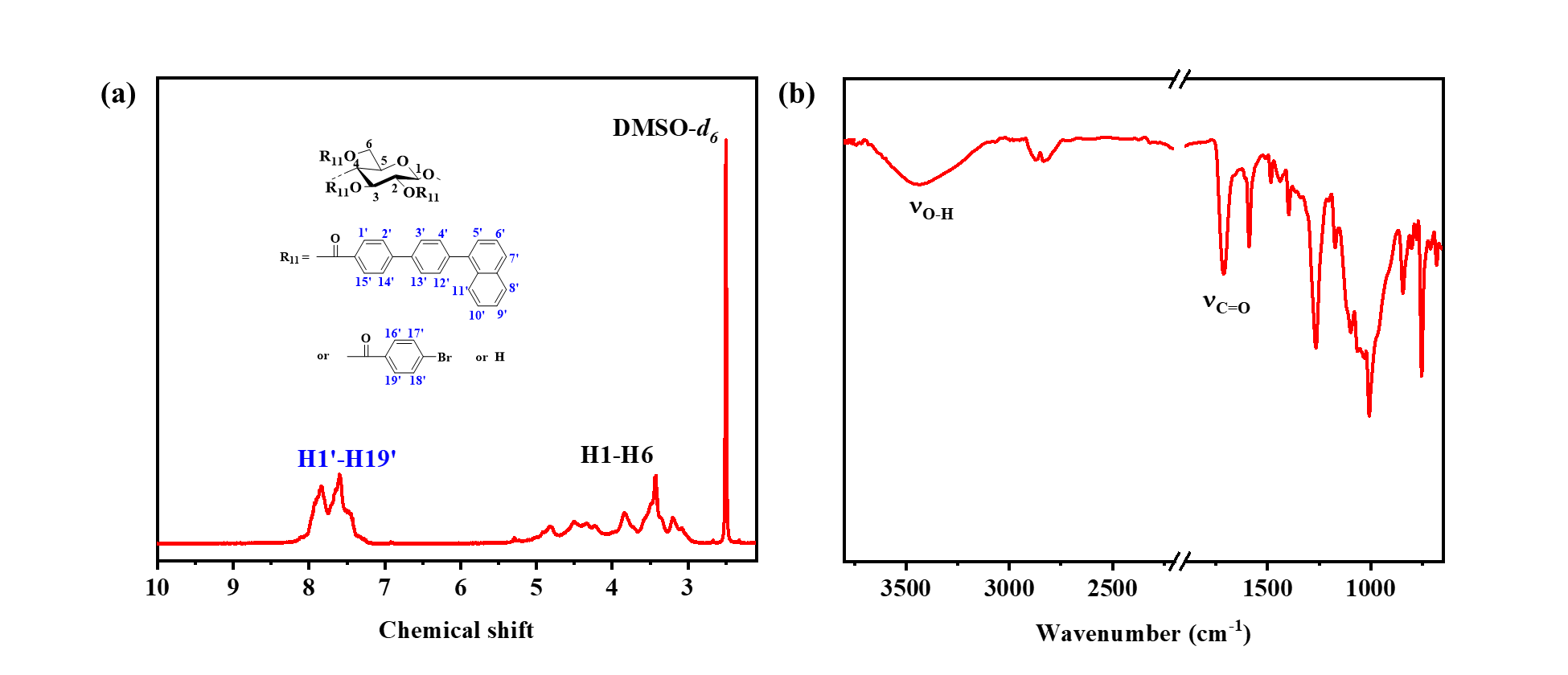


**Figure S12.** (a) ^1^H-NMR spectrum and (b) FTIR spectrum of CNBBA.


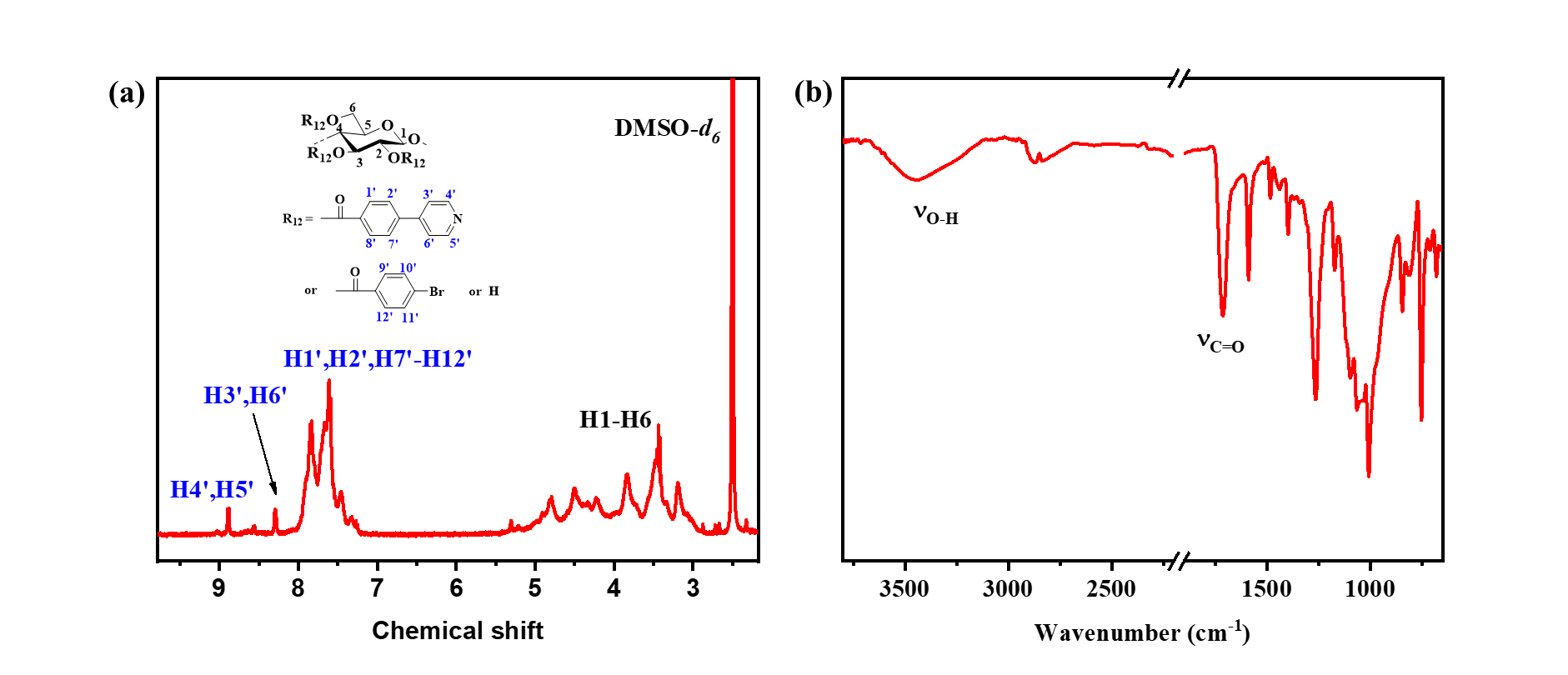


**Figure S13.** (a) ^1^H-NMR spectrum and (b) FTIR spectrum of CPYBA.


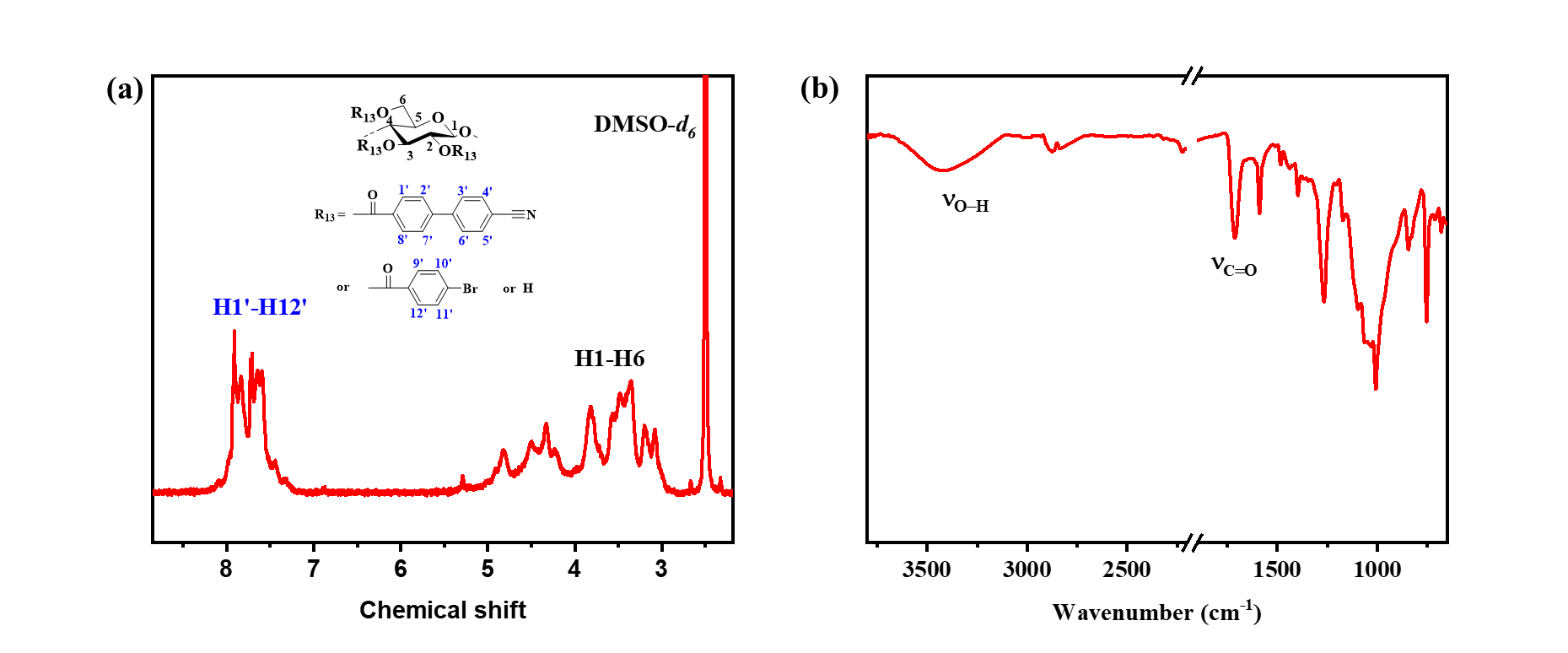


**Figure S14.** (a) ^1^H-NMR spectrum and (b) FTIR spectrum of CCPBA.


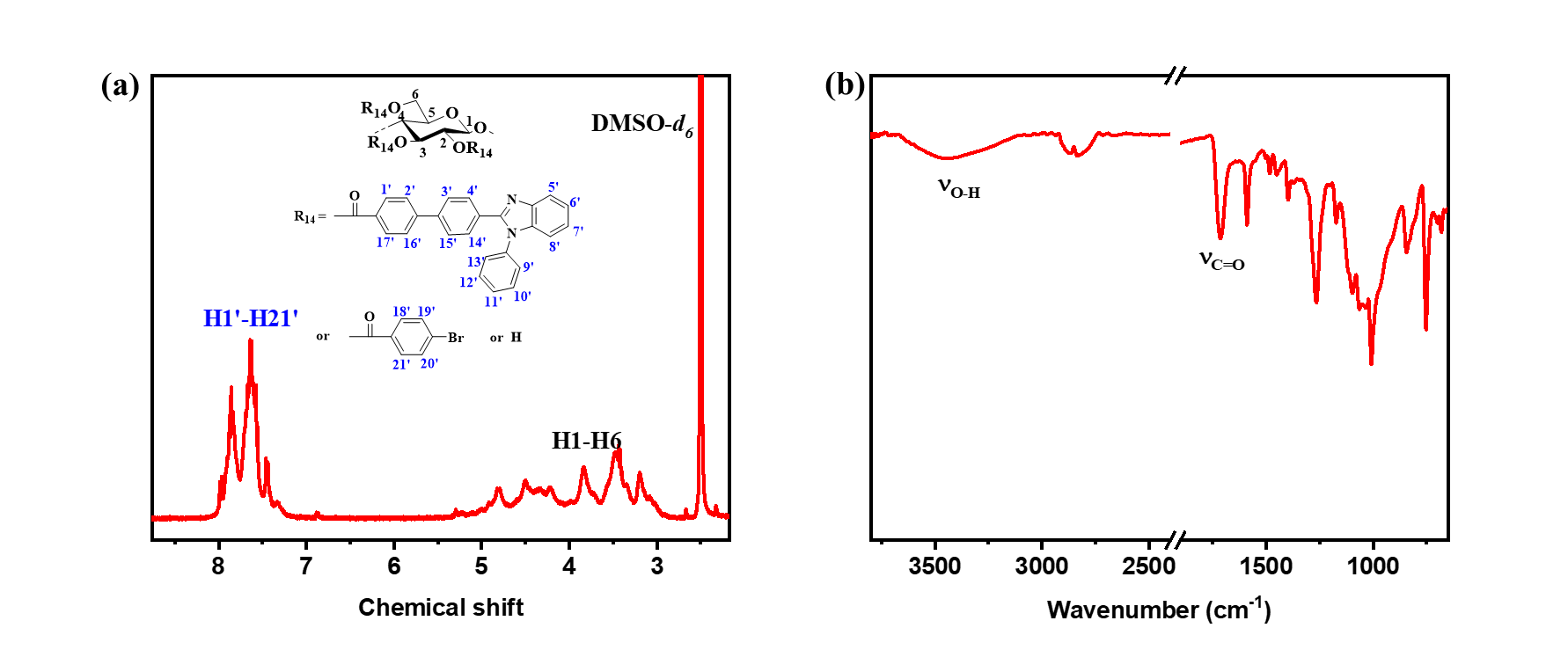


**Figure S15.** (a) ^1^H-NMR spectrum and (b) FTIR spectrum of CPBIPBA.

**Table S1.** CIE (x, y) coordinate values of CX@CNC.


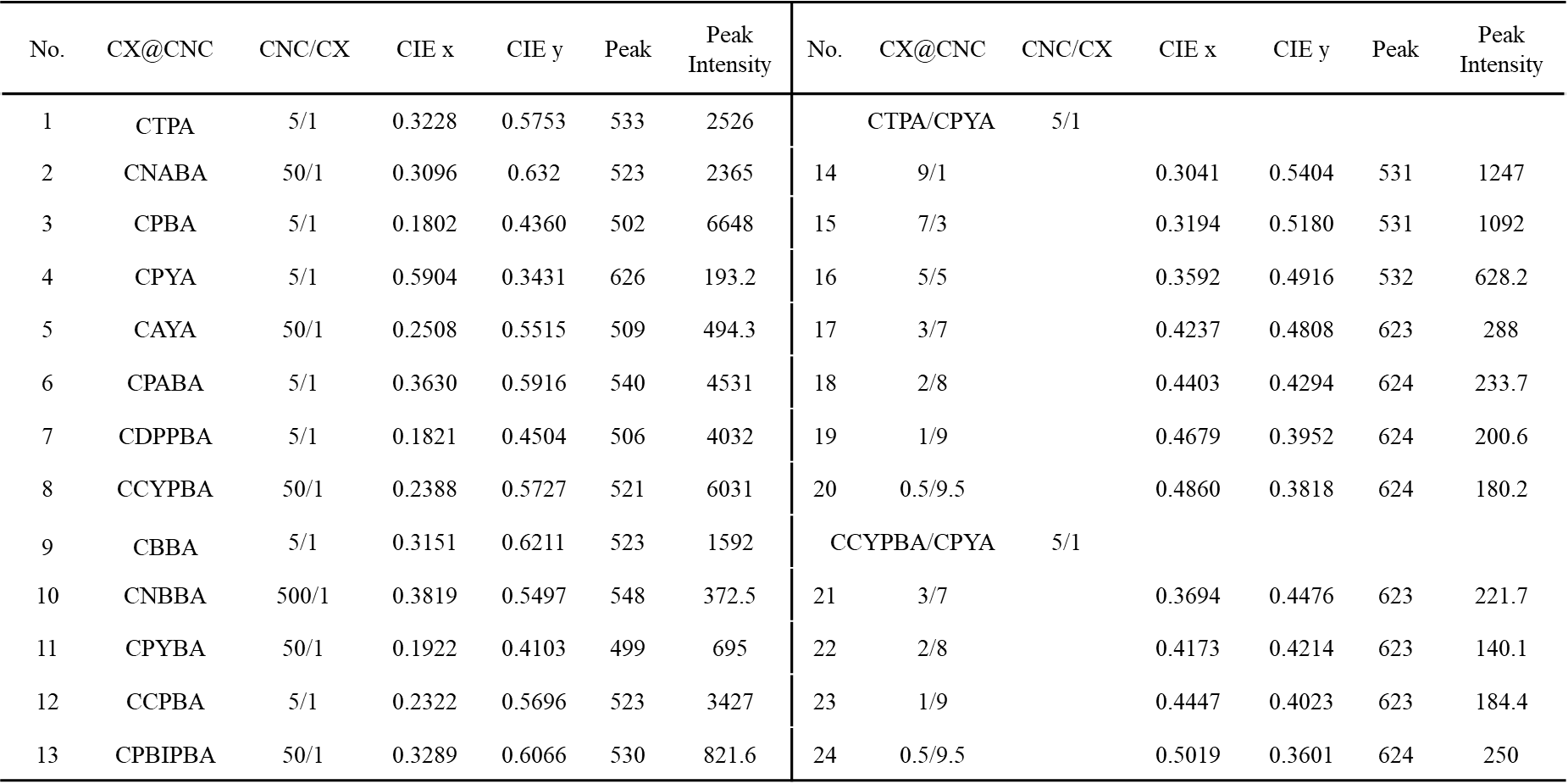


**Table S2.** Synthesis of CX under different reaction conditions.


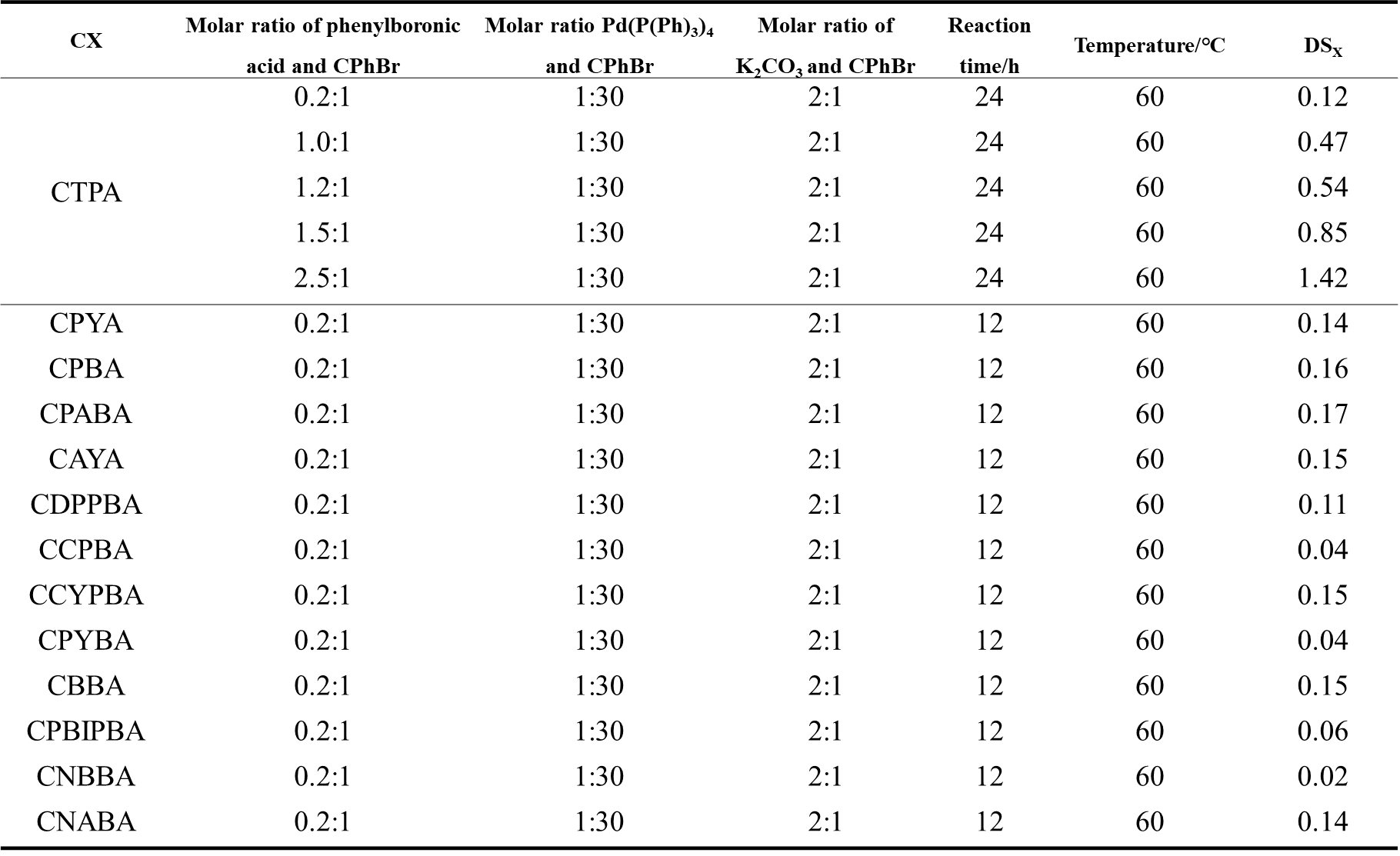


**Table S3.** RTP lifetime of CX@CNC.

**
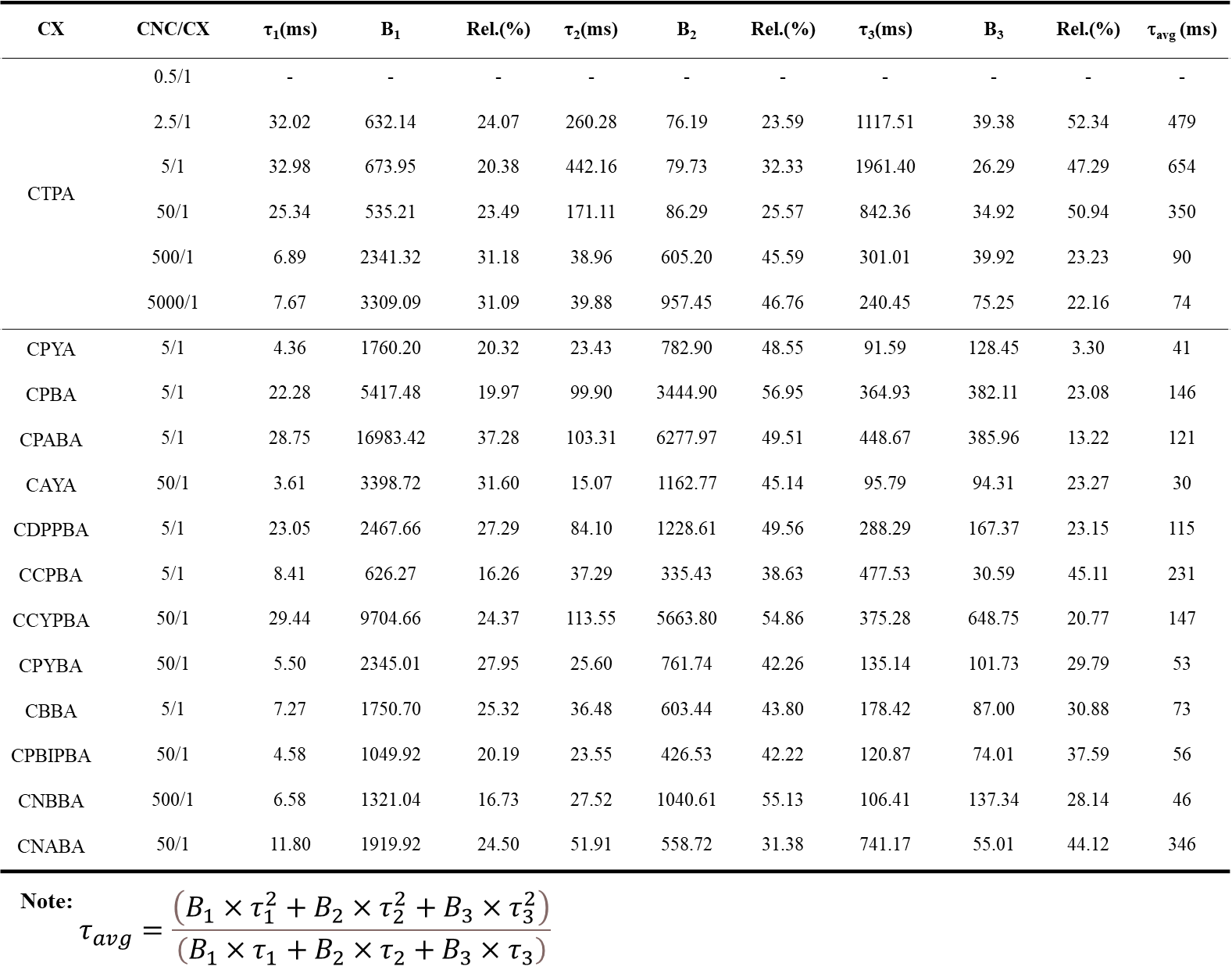
**

**Table S4.** λ_ex_, λ_em_, S_1_-S_0_, T_1_-S_0_, quantum yield and τ_avg_ of CX@CNC.


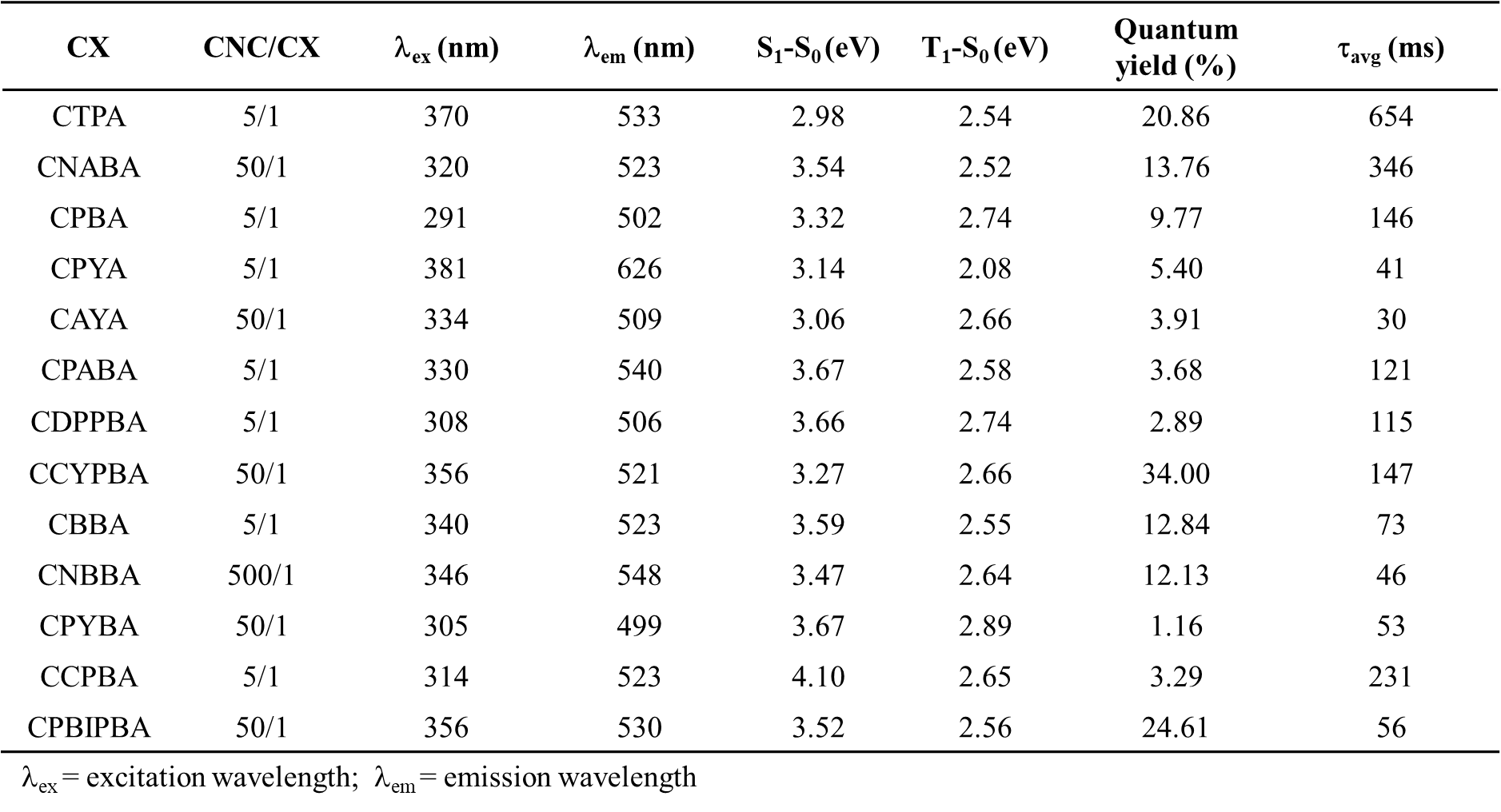


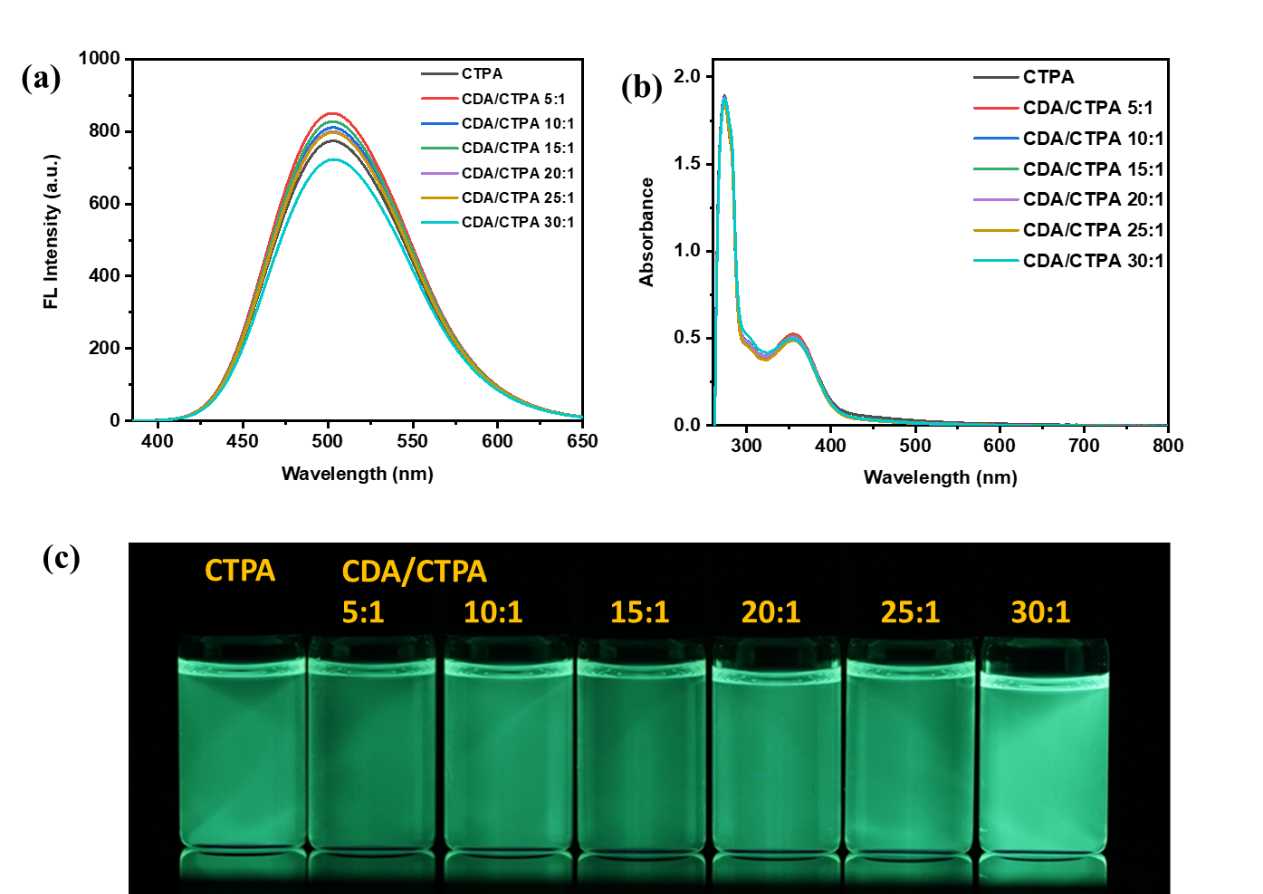


**Figure S16.** (a) Fluorescence spectra of CDA/CTPA/DMF solution with different mass ratios of CDA/CTPA (Ex = 365 nm). (b) Absorption spectra of CDA/CTPA/DMF solution with different mass ratios of CDA/CTPA . (c) Photographs of of CDA/CTPA/DMF solution with different mass ratios of CDA/CTPA taken under 365 nm UV light.

**Figure S17.** XRD curves of CNC and CTPA@CNC.


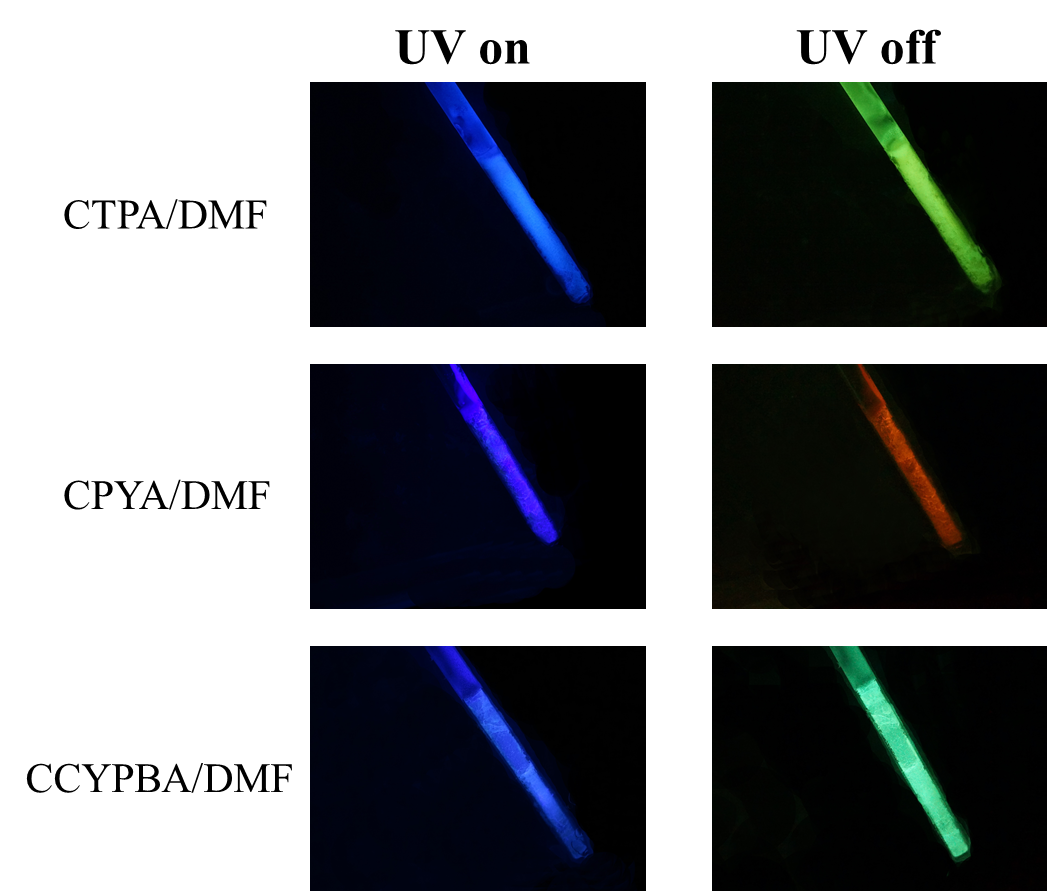


**Figure S18.** Photographs of fluorescence and phosphorescence of CX/DMF solutions (0.1 mol/L) at 77 K.


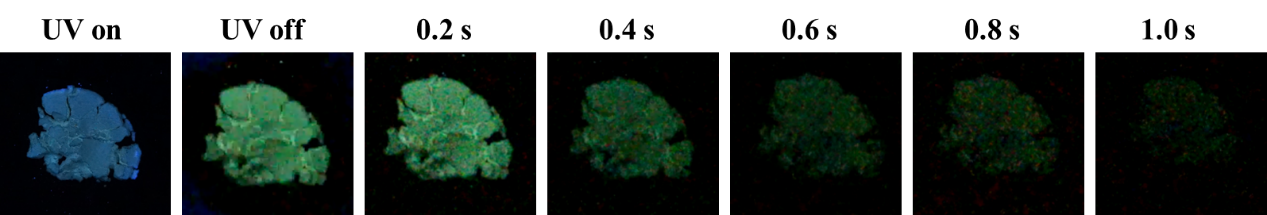


**Figure S19.** Photographs of fluorescence and phosphorescence of cellulose powder at room temperature.


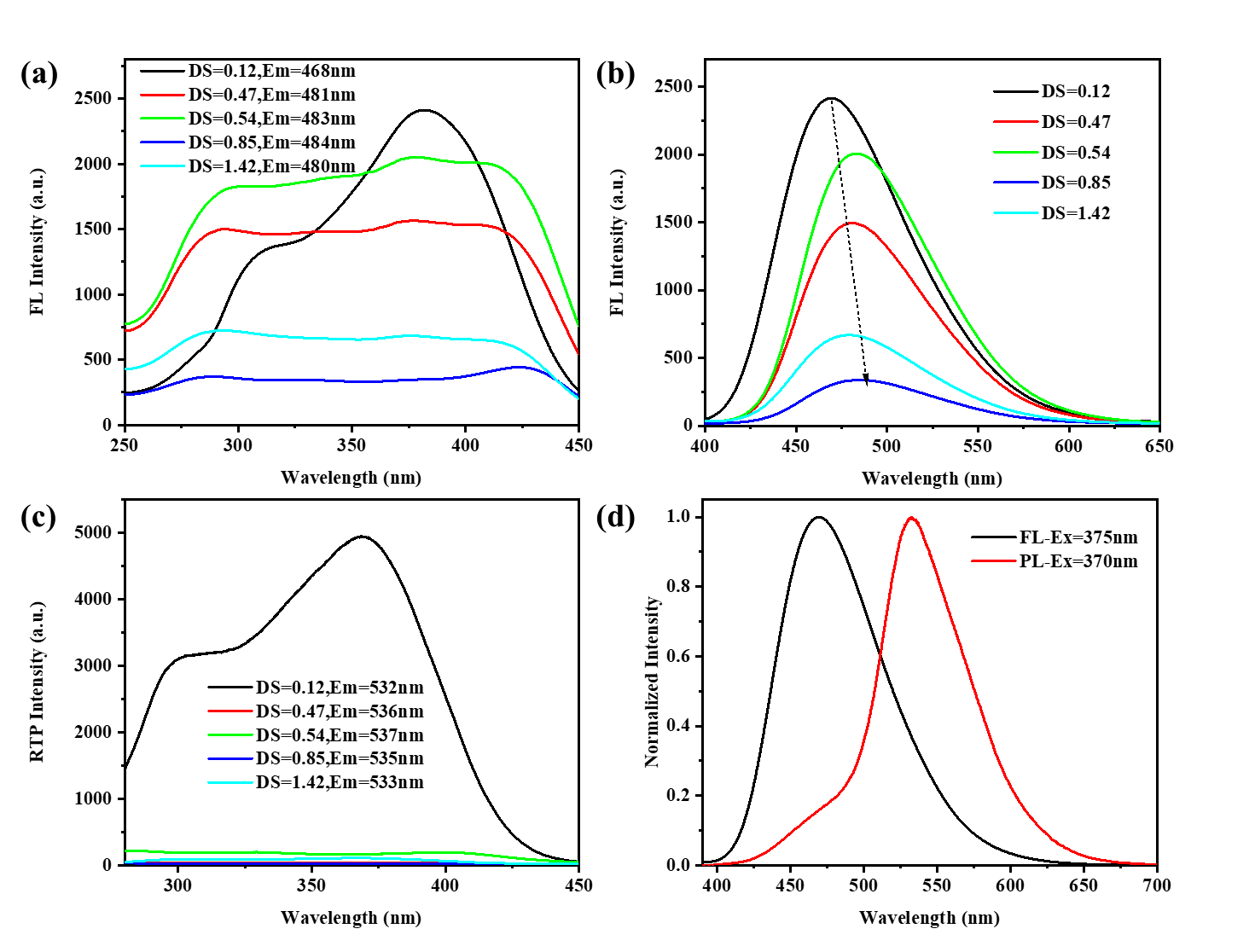


**Figure S20.** (a) Fluorescence excitation spectra, (b) Fluorescence emission spectra (Ex = 375 nm), (c) Phosphorescence excitation spectra of CTPA@CNC (CNC/CTPA = 5:1 (w/w)) with different DS of CTPA. (d) Fluorescence spectrum and phosphorescence spectrum of CTPA@CNC (DS_TPA_ = 0.12; CNC/CTPA = 5:1 (w/w)).


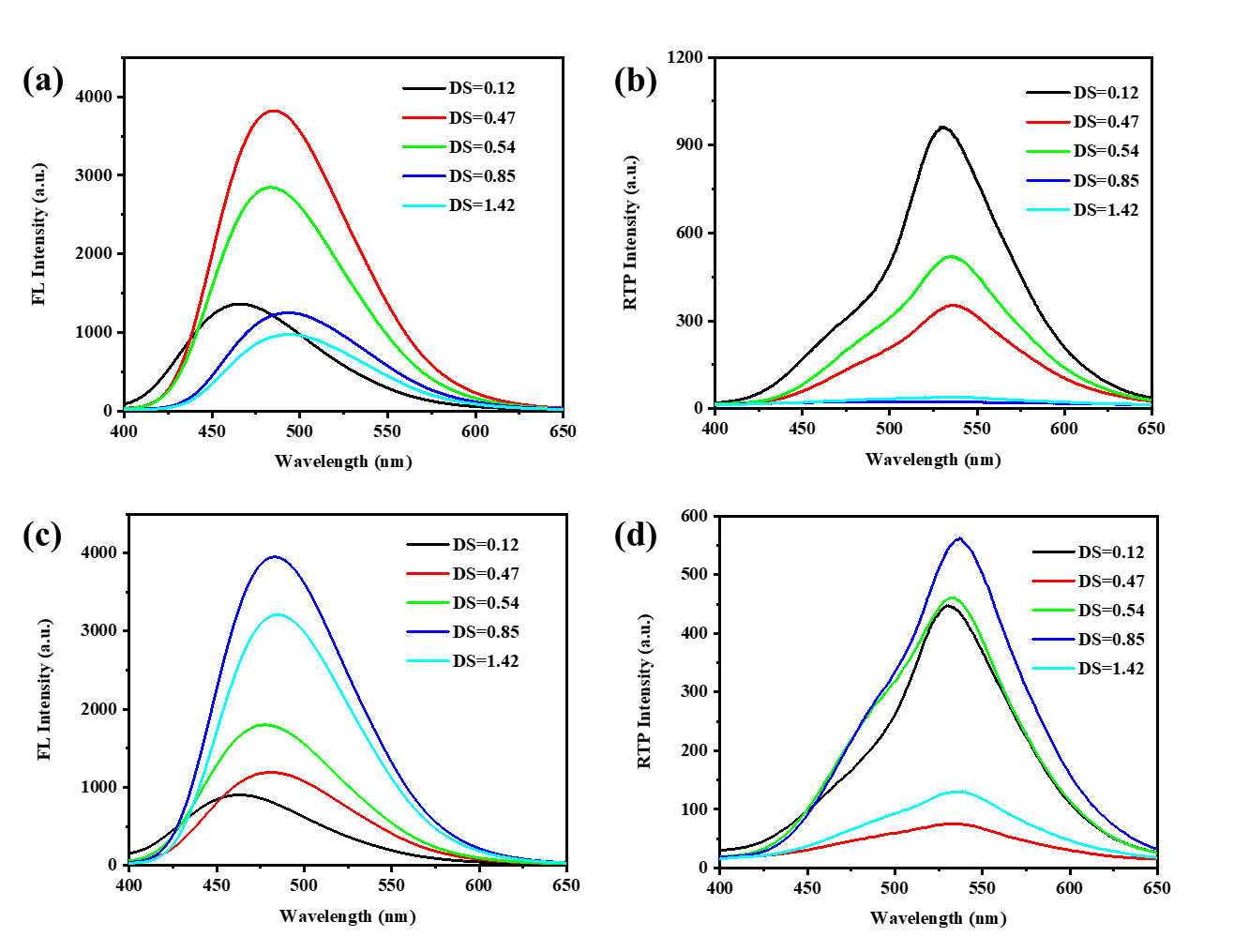


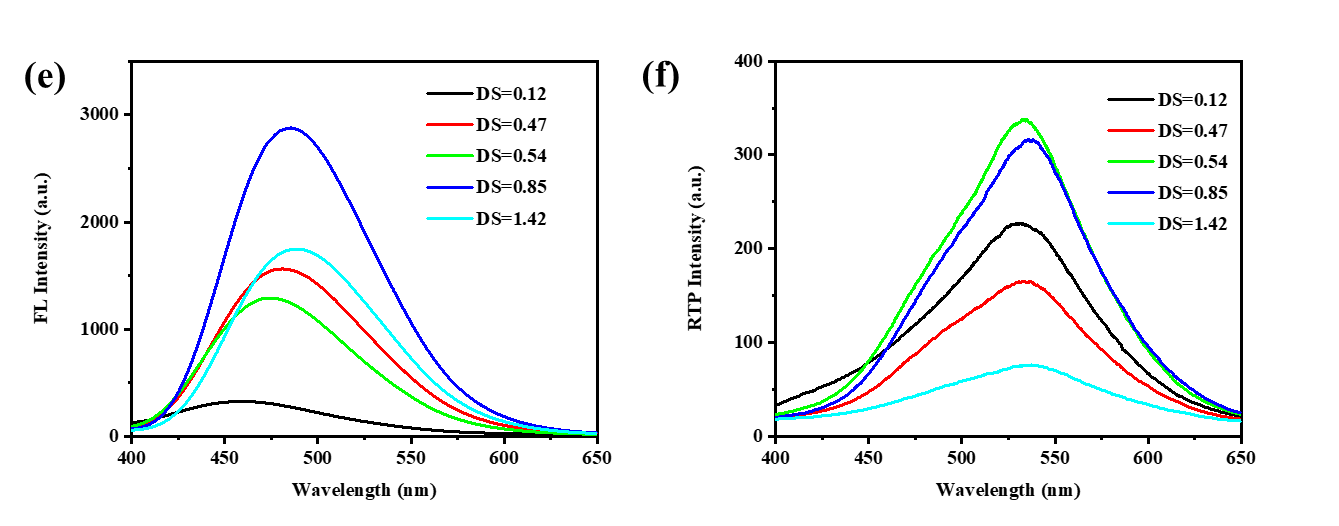


**Figure S21.** (a) Fluorescence emission spectra (Ex = 375 nm) and (b) Phosphorescence spectra (Ex = 370 nm) of CTPA@CNC (CNC/CTPA = 50/1) with different DS of CTPA. (c) Fluorescence emission spectra (Ex = 375 nm) and (d) Phosphorescence spectra (Ex = 370 nm) of CTPA@CNC (CNC/CTPA = 500/1) with different DS of CTPA. (e) Fluorescence emission spectra (Ex = 375 nm) and (b) Phosphorescence spectra (Ex = 370 nm) of CTPA@CNC (CNC/CTPA = 5000/1) with different DS of CTPA.


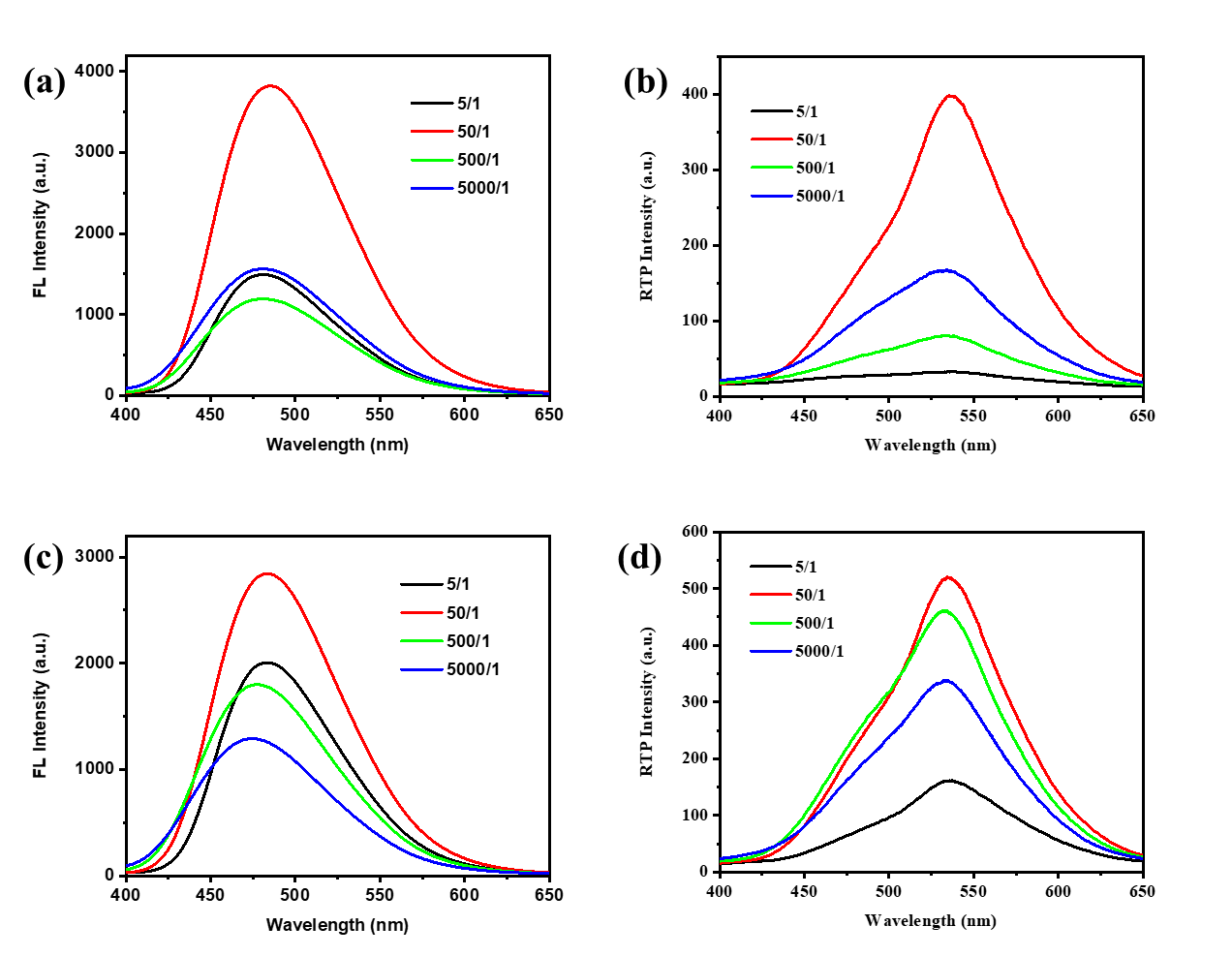


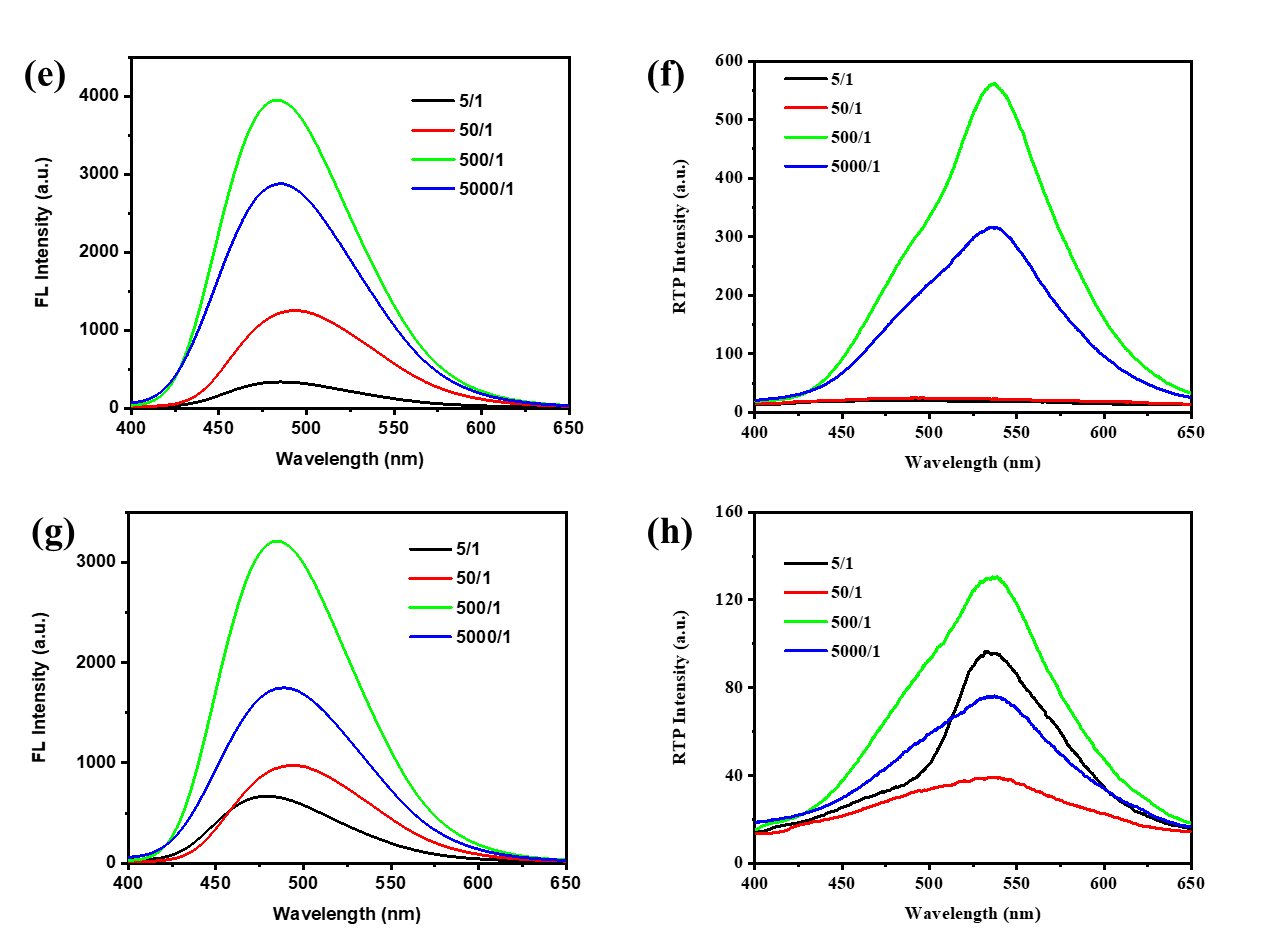


**Figure S22.** (a) Fluorescence emission spectra (Ex = 375 nm) and (b) Phosphorescence emission spectra (Ex = 370 nm) of CTPA@CNC (DS_TPA_ = 0.47) with different CNC/CTPA ratio. (c) Fluorescence emission spectra (Ex = 375 nm) and (d) Phosphorescence emission spectra (Ex = 370 nm) of CTPA@CNC (DS_TPA_ = 0.54) with different CNC/CTPA ratio. (e) Fluorescence emission spectra (Ex = 375 nm) and (f) Phosphorescence emission spectra (Ex = 370 nm) of CTPA@CNC (DS_TPA_ = 0.85) with different CNC/CTPA ratio. (g) Fluorescence emission spectra (Ex = 375 nm) and (h) Phosphorescence emission spectra (Ex = 370 nm) of CTPA@CNC (DS_TPA_ = 1.42) with different CNC/CTPA ratio.


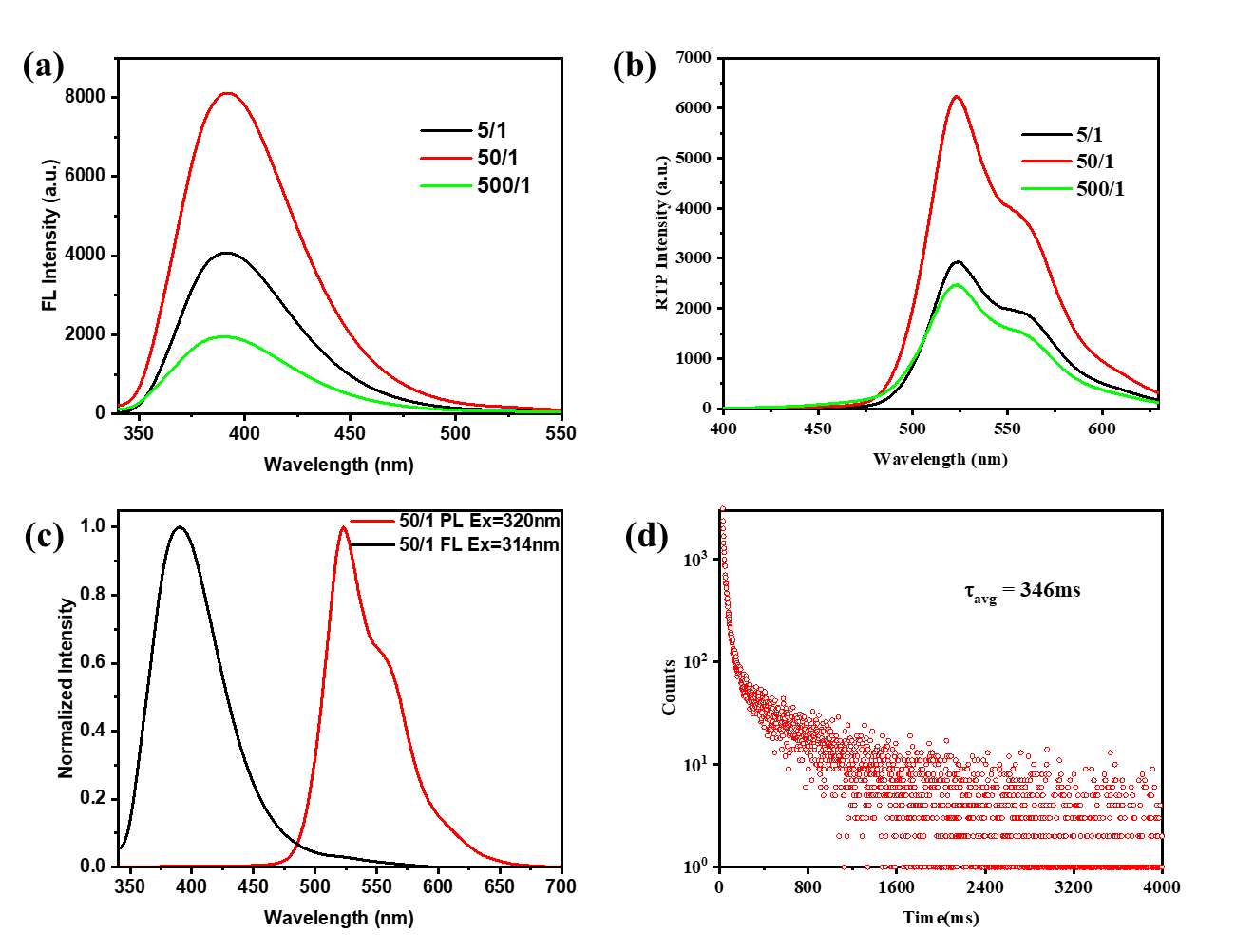


**Figure S23.** (a) Fluorescence emission spectra (Ex = 320 nm) and (b) Phosphorescence emission spectra (Ex = 314 nm) of CNABA@CNC with different mass ratios of CNC/CNABA. (c) Fluorescence spectrum and phosphorescence spectrum, and (d) RTP lifetime spectrum of CNABA@CNC (CNC/CNABA = 50/1).


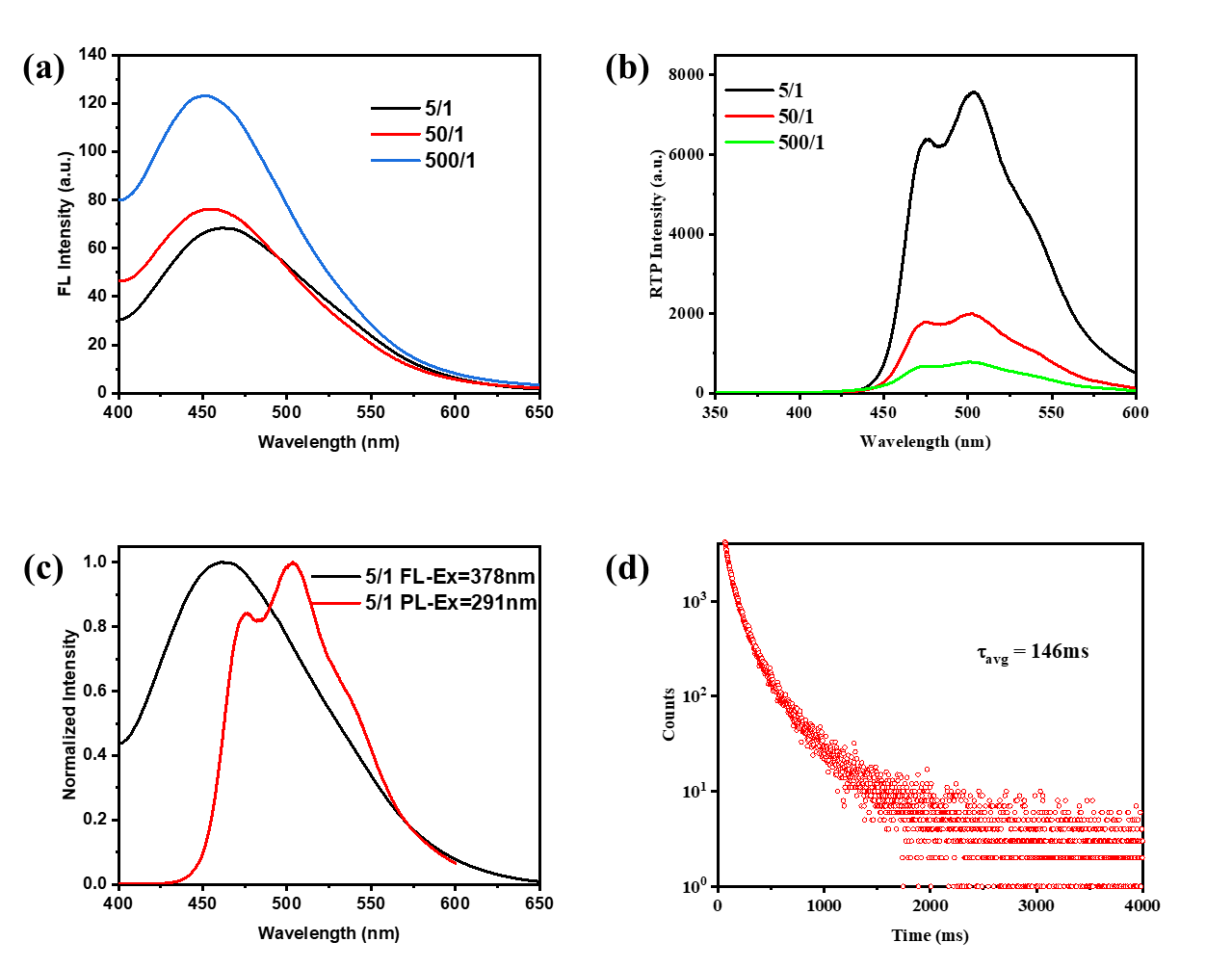


**Figure S24.** (a) Fluorescence emission spectra (Ex = 378 nm) and (b) Phosphorescence emission spectra (Ex = 291 nm) of CPBA@CNC with different mass ratios of CNC/CPBA. (c) Fluorescence spectrum and phosphorescence spectrum, and (d) RTP lifetime spectrum of CPBA@CNC (CNC/CPBA = 5/1).


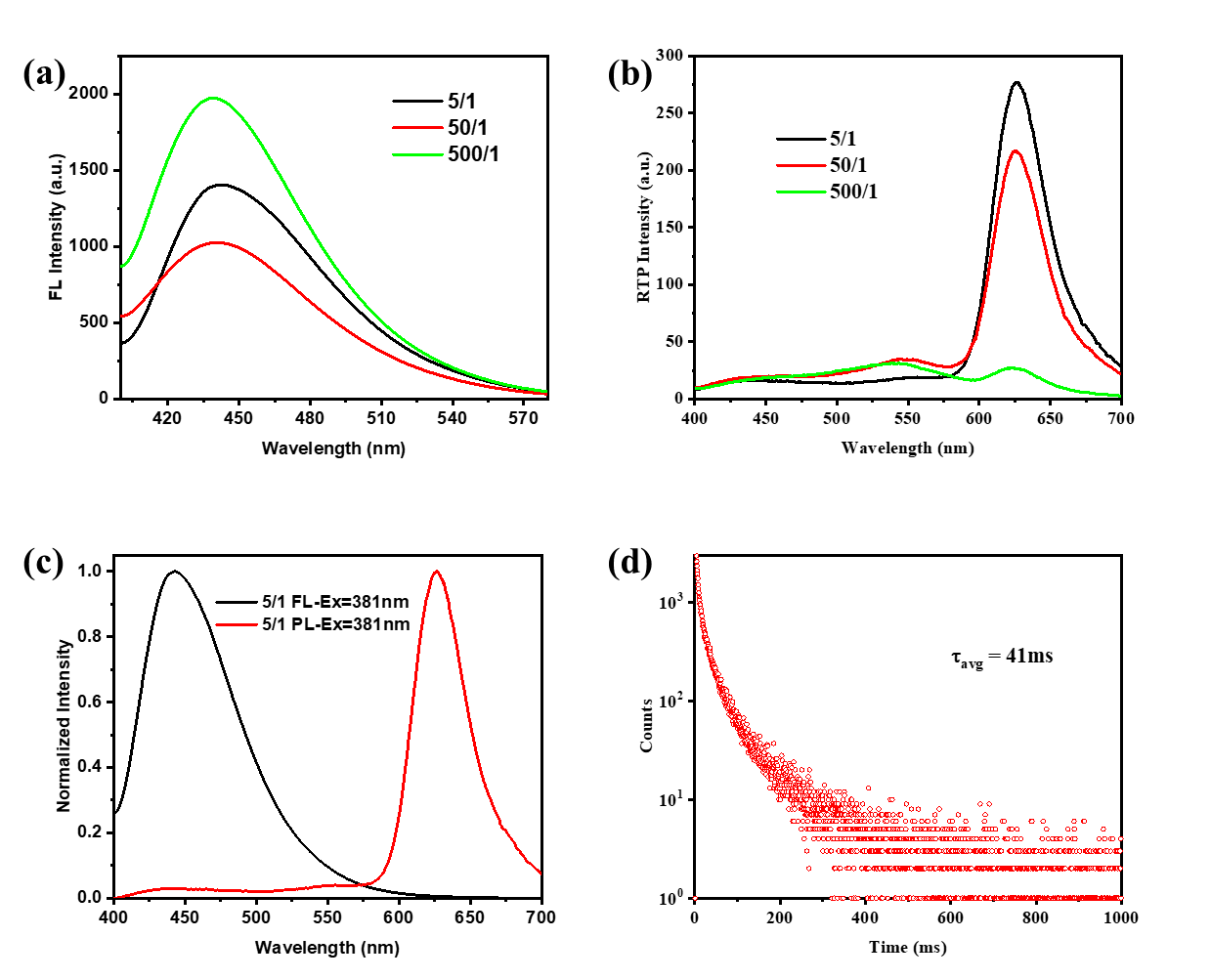


**Figure S25.** (a) Fluorescence emission spectra (Ex = 381 nm) and (b) Phosphorescence emission spectra (Ex = 381 nm) of CPYA@CNC with different mass ratios of CNC/CPYA. (c) Fluorescence spectrum and phosphorescence spectrum, and (d) RTP lifetime spectrum of CPYA@CNC (CNC/CPYA = 5/1).


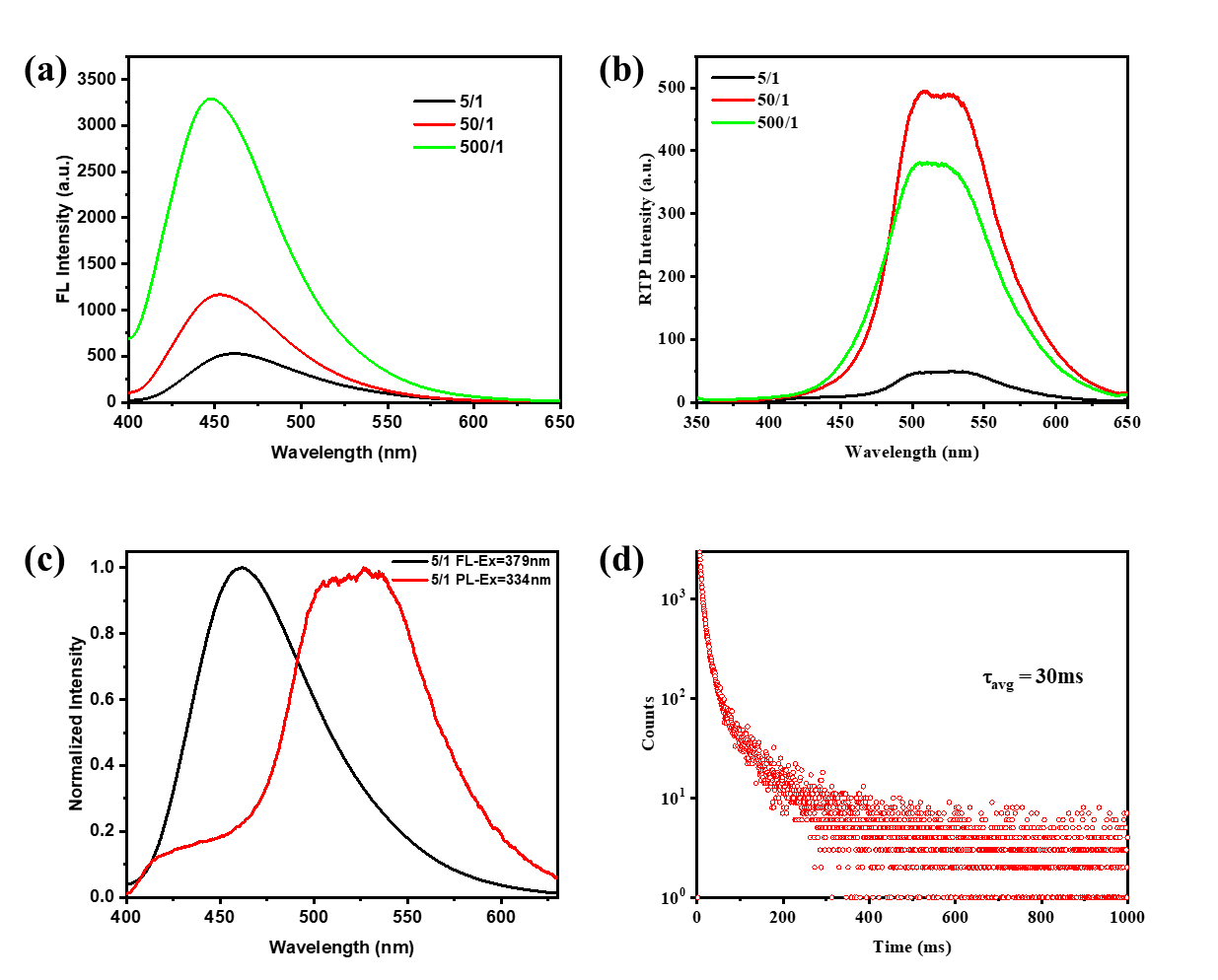


**Figure S26.** (a) Fluorescence emission spectra (Ex = 379 nm) and (b) Phosphorescence emission spectra (Ex = 334 nm) of CAYA@CNC with different mass ratios of CNC/CAYA. (c) Fluorescence spectrum and phosphorescence spectrum, and (d) RTP lifetime spectrum of CAYA@CNC (CNC/CAYA = 50/1).


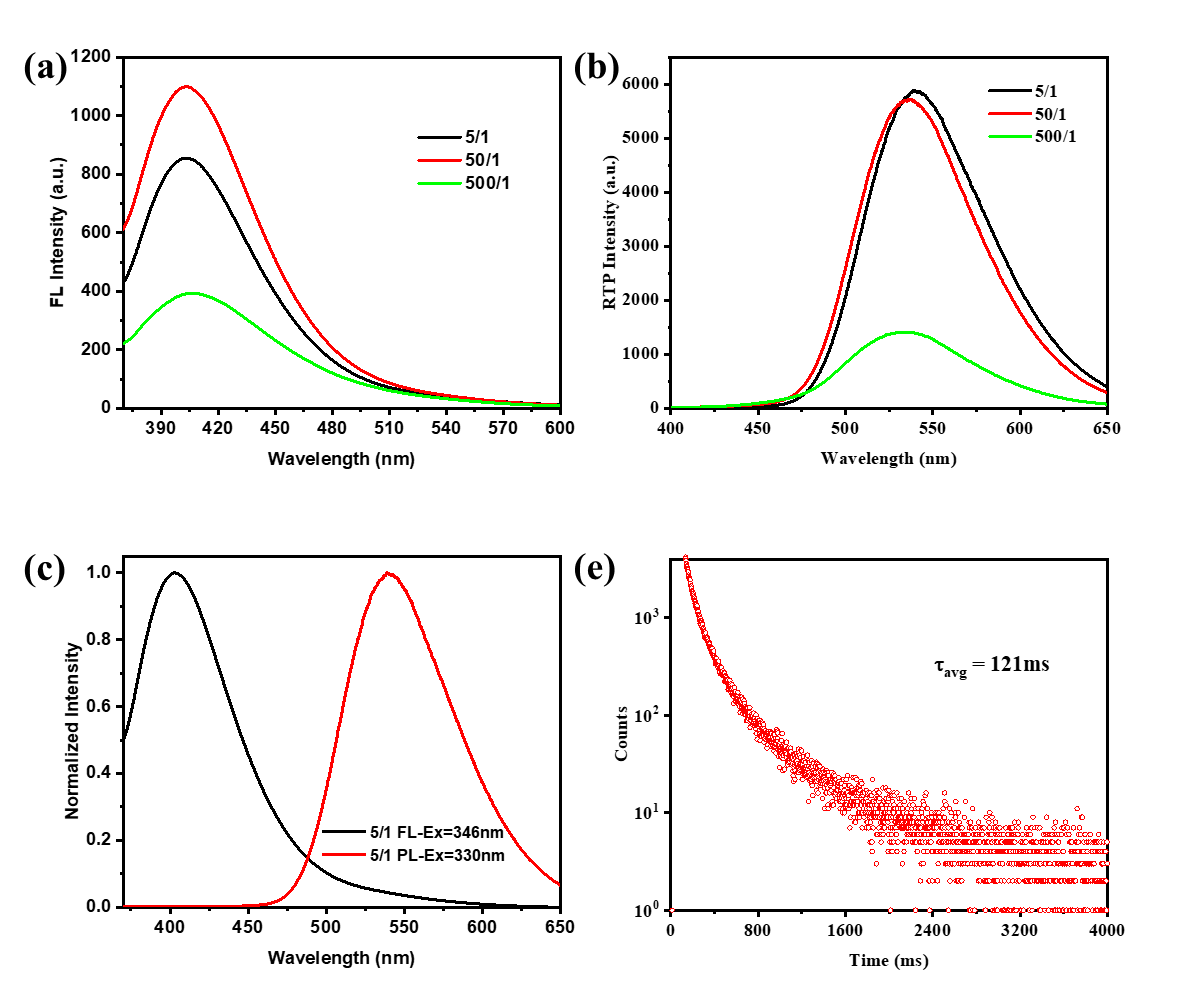


**Figure S27.** (a) Fluorescence emission spectra (Ex = 346 nm) and (b) Phosphorescence emission spectra (Ex = 330 nm) of CPABA@CNC with different mass ratios of CNC/CPABA. (c) Fluorescence spectrum and phosphorescence spectrum, and (d) RTP lifetime spectrum of CPABA@CNC (CNC/CPABA = 5/1).


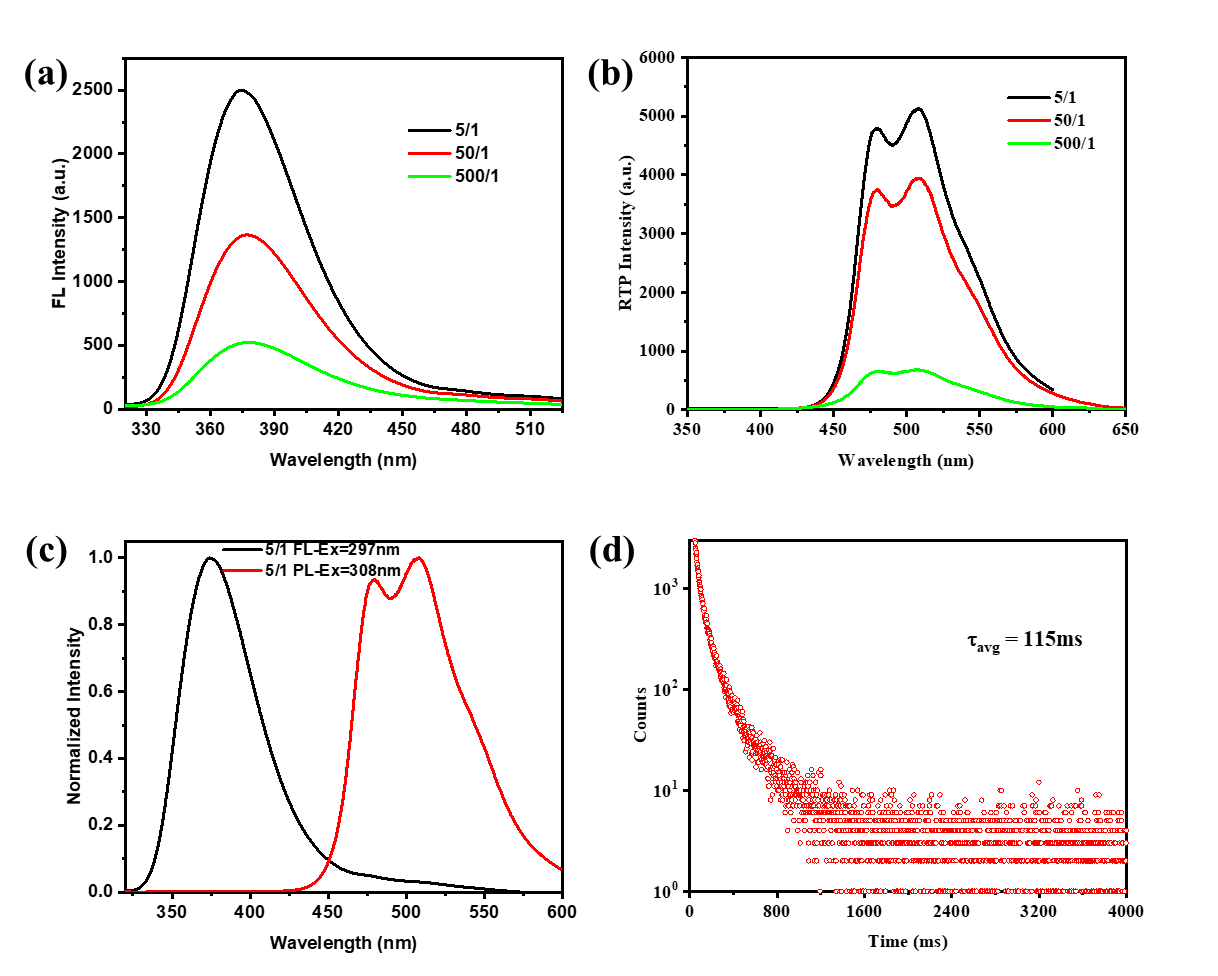


**Figure S28.** (a) Fluorescence emission spectra (Ex = 297 nm) and (b) Phosphorescence emission spectra (Ex = 308 nm) of CDPPBA@CNC with different mass ratios of CNC/CDPPBA. (c) Fluorescence spectrum and phosphorescence spectrum, and (d) RTP lifetime spectrum of CDPPBA@CNC (CNC/CDPPBA = 5/1).


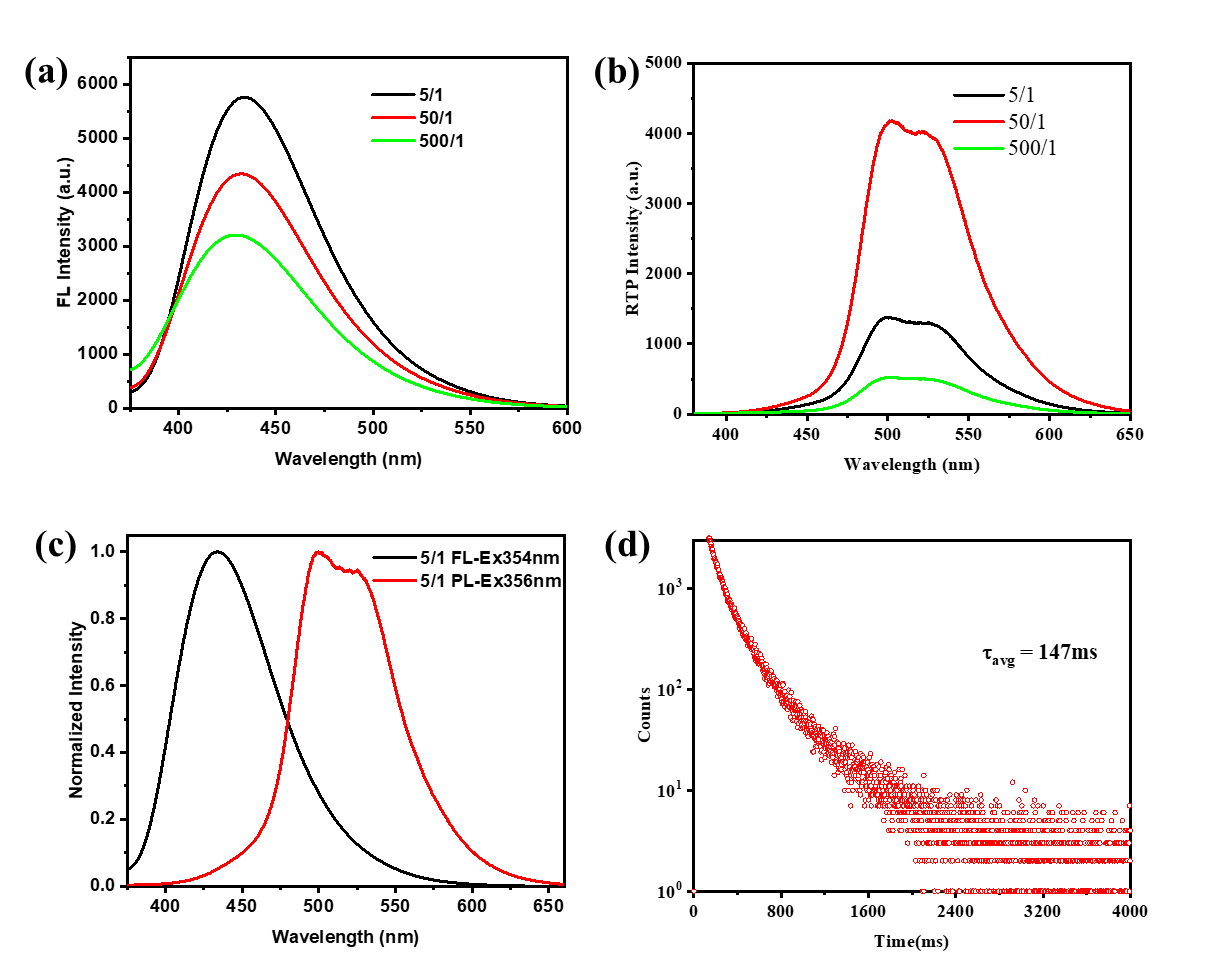


**Figure S29.** (a) Fluorescence emission spectra (Ex = 354 nm) and (b) Phosphorescence emission spectra (Ex = 356 nm) of CCYPBA@CNC with different mass ratios of CNC/CCYPBA. (c) Fluorescence spectrum and phosphorescence spectrum, and (d) RTP lifetime spectrum of CCYPBA@CNC (CNC/CCYPBA = 50/1).


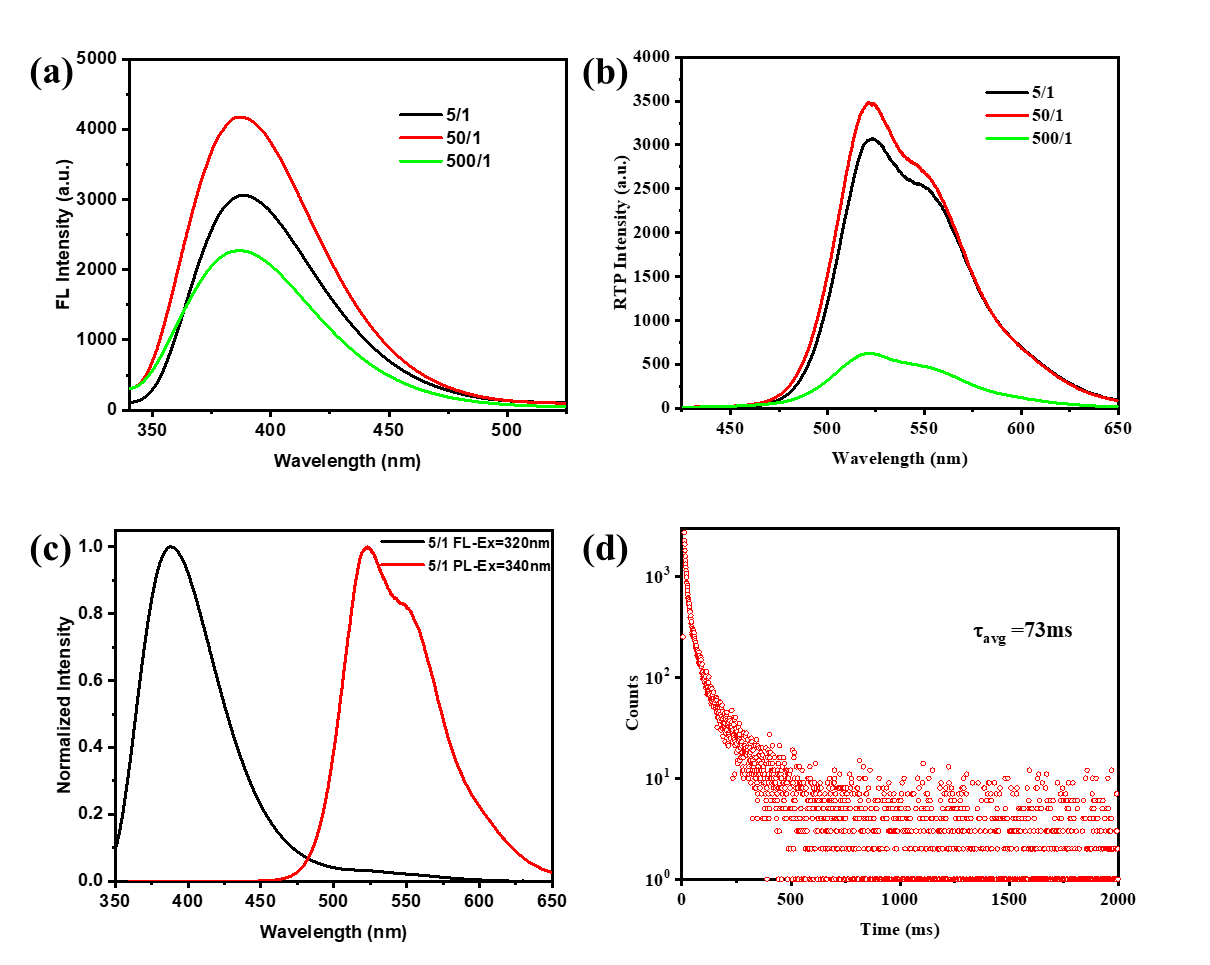


**Figure S30.** (a) Fluorescence emission spectra (Ex = 320 nm) and (b) Phosphorescence emission spectra (Ex = 340 nm) of CBBA@CNC with different mass ratios of CNC/CBBA. (c) Fluorescence spectrum and phosphorescence spectrum, and (d) RTP lifetime spectrum of CBBA@CNC (CNC/CBBA = 5/1).


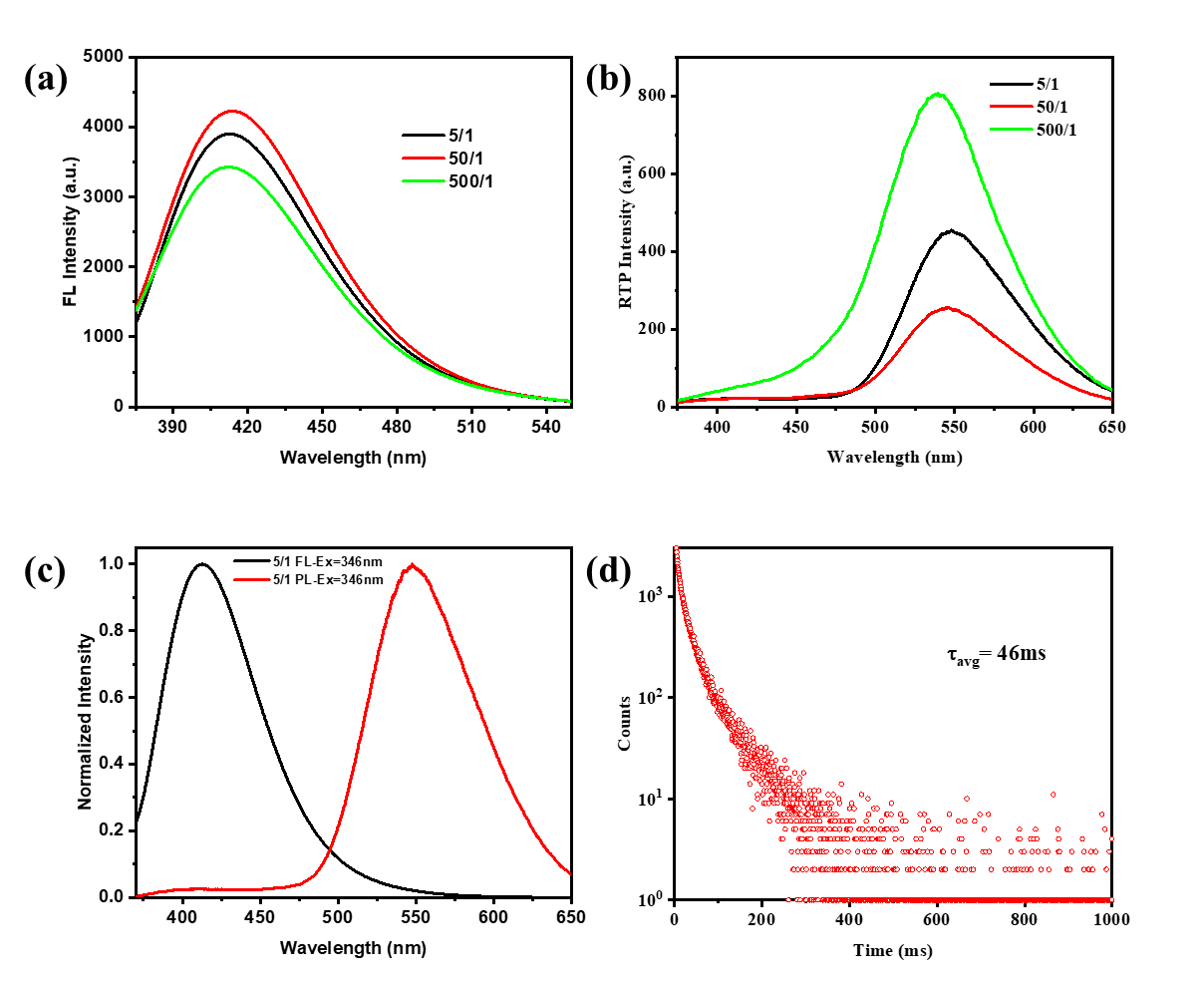


**Figure S31.** (a) Fluorescence emission spectra (Ex = 346 nm) and (b) Phosphorescence emission spectra (Ex = 346 nm) of CNBBA@CNC with different mass ratios of CNC/CNBBA. (c) Fluorescence spectrum and phosphorescence spectrum, and (d) RTP lifetime spectrum of CNBBA@CNC (CNC/CNBBA = 500/1).


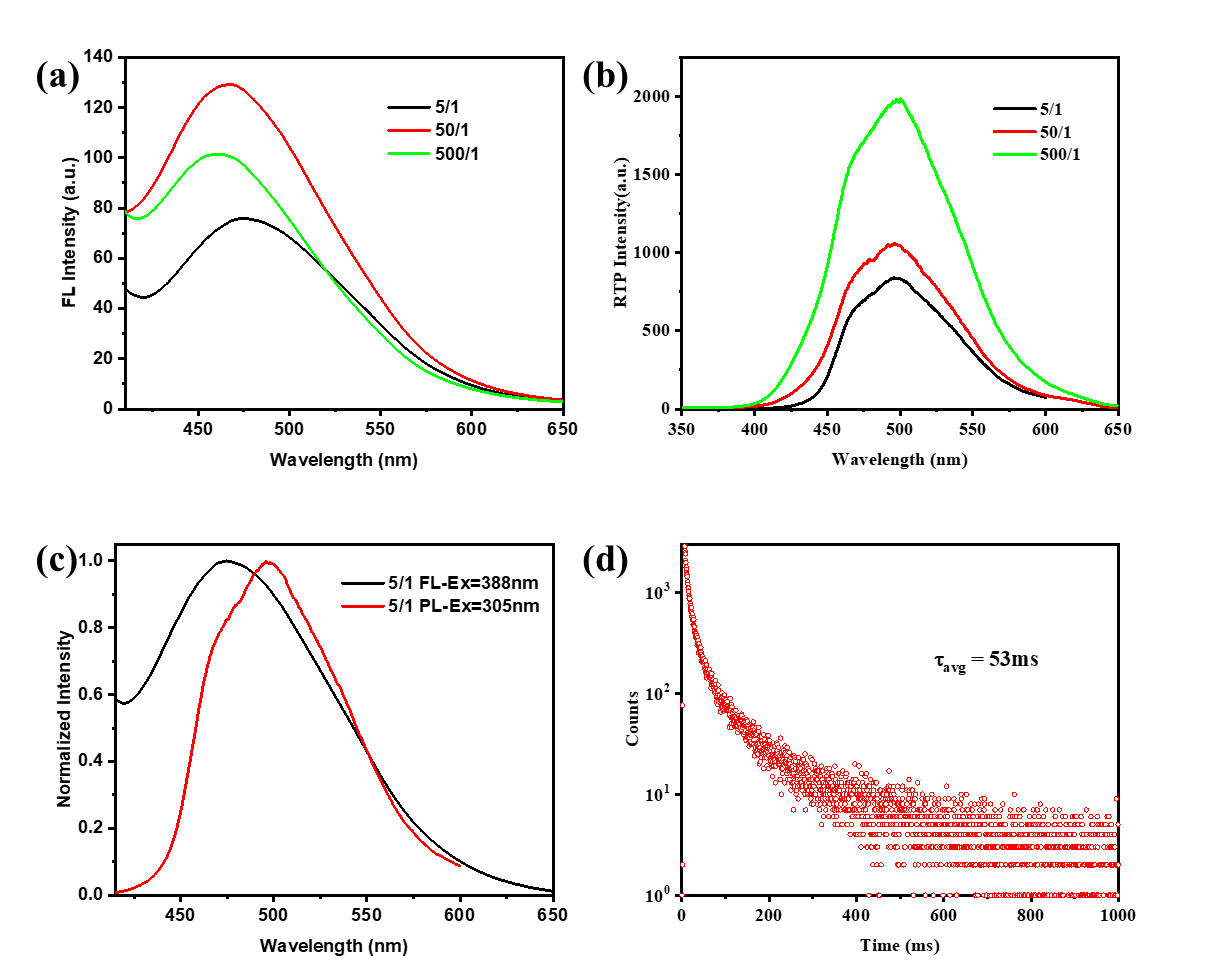


**Figure S32.** (a) Fluorescence emission spectra (Ex = 388 nm) and (b) Phosphorescence emission spectra (Ex = 305 nm) of CPYBA@CNC with different mass ratios of CNC/CPYBA. (c) Fluorescence spectrum and phosphorescence spectrum, and (d) RTP lifetime spectrum of CPYBA@CNC (CNC/CPYBA = 50/1).


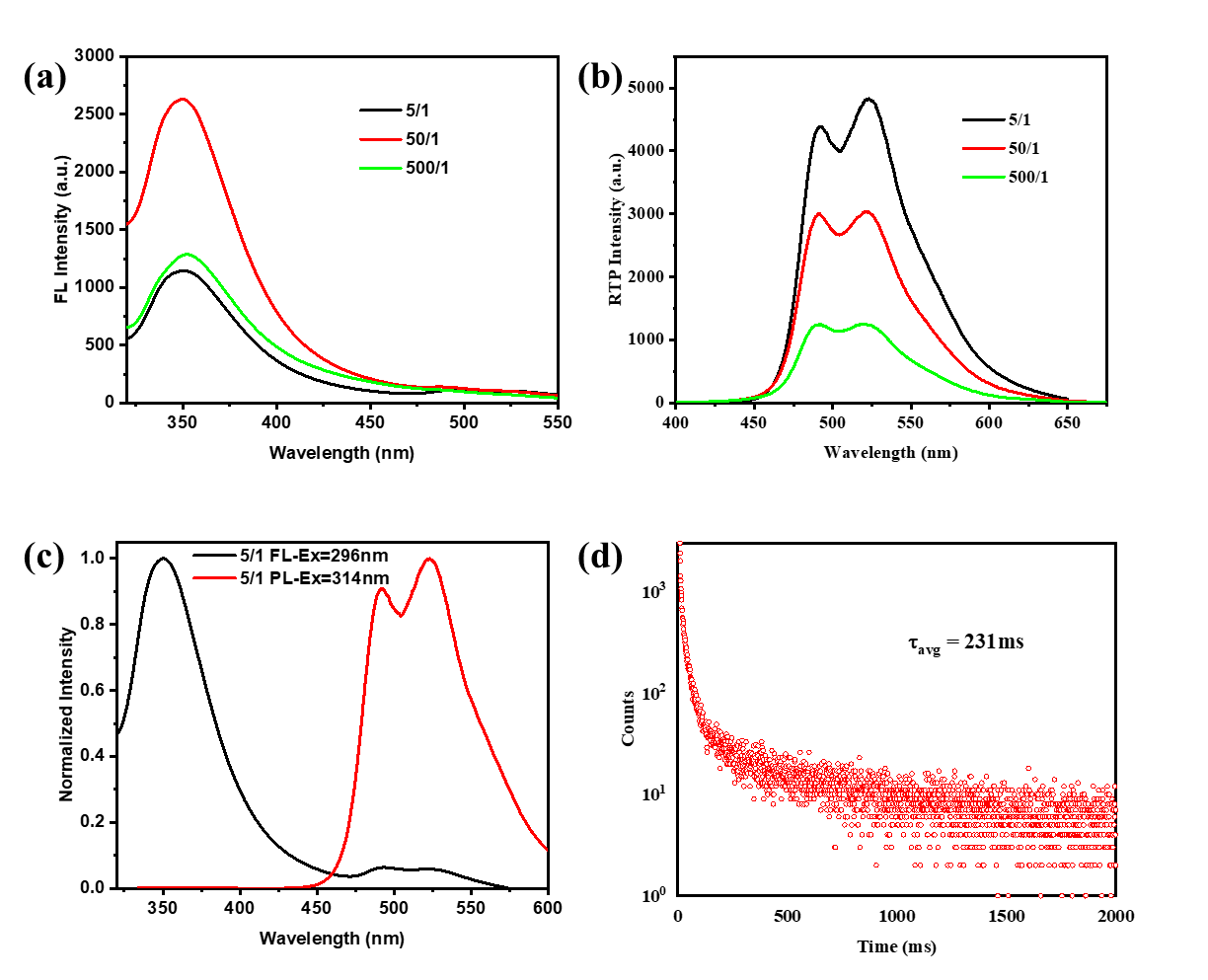


**Figure S33.** (a) Fluorescence emission spectra (Ex = 296 nm) and (b) Phosphorescence emission spectra (Ex = 314 nm) of CCPBA@CNC with different mass ratios of CNC/CCPBA. (c) Fluorescence spectrum and phosphorescence spectrum, and (d) RTP lifetime spectrum of CCPBA@CNC (CNC/CCPBA = 5/1).


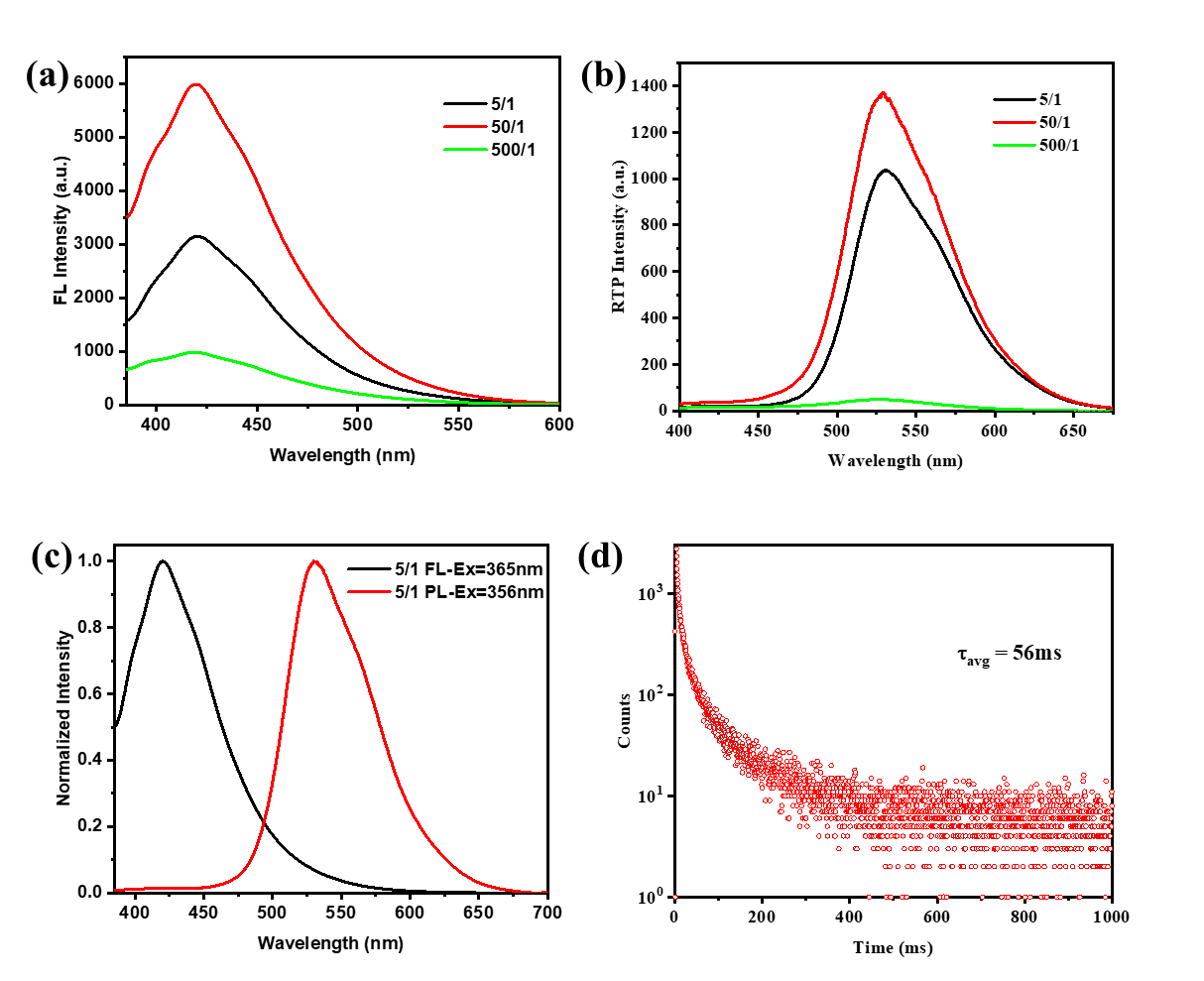


**Figure S34.** (a) Fluorescence emission spectra (Ex = 365 nm) and (b) Phosphorescence emission spectra (Ex = 356 nm) of CPBIPBA@CNC with different mass ratios of CNC/CPBIPBA. (c) Fluorescence spectrum and phosphorescence spectrum, and (d) RTP lifetime spectrum of CPBIPBA@CNC (CNC/CPBIPBA = 50/1).


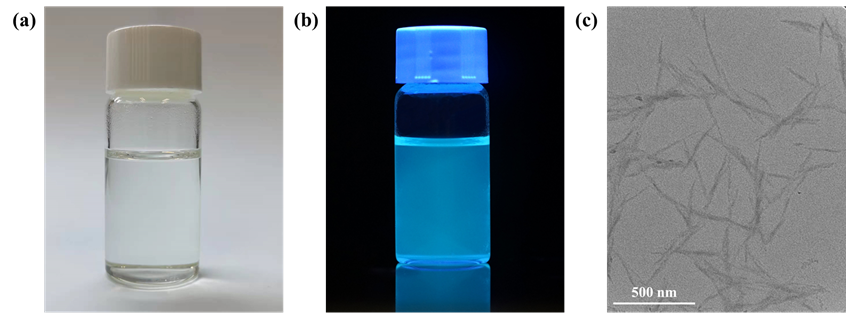


**Figure S35.** (a) Photograph under visible light, (b) Fluorescent photograph under 365-nm ultraviolet light, and (c) TEM image of CTPA@CNC in DMF solution. (CNC/CTPA = 5:1; CNC, 0.1 wt%)

**References**

[1] H. Zhang, J. Wu, J. Zhang, and J.S. He, “1-Allyl-3-methylimidazolium Chloride Room Temperature Ionic Liquid:  A New and Powerful Nonderivatizing Solvent for Cellulose,” *Macromolecules*, vol. *38*, pp. 8272-8277, 2005.
